# The dark sides of the brain: A systematic review and meta-analysis of functional neuroimaging studies on trait aggression^☆,☆☆^

**DOI:** 10.1016/j.avb.2025.102035

**Published:** 2025-02-05

**Authors:** Jules R. Dugré, Christian J. Hopfer, Drew E. Winters

**Affiliations:** a School of Psychology and Centre for Human Brain Health, University of Birmingham, Birmingham, UK; b Department of Psychiatry, University of Colorado School of Medicine, Anschutz Medical Campus, CO, USA

**Keywords:** Meta-analysis, Neuroimaging, Aggression, Reactive aggression, Proactive aggression

## Abstract

Aggression is a worldwide issue that has significant consequences for both the victims and societies. However, aggression may vary in its underlying motivation (i.e., reactive versus proactive) and the forms in which it occurs (i.e., physical versus verbal). Yet, functional brain correlates differentiating these types remains largely unknown. A systematic search was conducted up to May 1st 2023, using PubMed, Google Scholar, and Web of Science, to identify relevant functional neuroimaging studies that included measures of General Aggression, Reactive Aggression, Proactive Aggression, Physical Aggression and Verbal Aggression. Coordinate-based meta-analysis was conducted using both spatial convergence (ALE) and effect-size (SDM-PSI) approaches. Sixty-seven functional neuroimaging studies met the inclusion criteria. Meta-analysis revealed similar yet distinct neural correlates for General Aggression (i.e., Amygdala, Precuneus, Intraparietal Sulcus, Angular and Middle Temporal Gyri), Reactive Aggression (i.e., Amygdala, Periaqueductal Grey, Posterior Insula, & Central Opercular Cortex), Proactive Aggression (i.e., Septal Area, & Amygdala), Physical Aggression (i.e., Dorsal Premotor Cortex, Dorsal Caudate, & Dorsal Anterior Cingulate Cortex), and Verbal (i.e., Dorsal Anterior Cingulate Cortex). Exploratory analyses revealed the importance of affective, cognitive and social cognition processes as well as serotoninergic, dopaminergic, and cholinergic systems in the neural underpinnings of aggressive behaviors. Our findings highlight the importance of examining the types of aggression (i.e., motivation and forms) within a transdiagnostic framework. Therefore, characterizing the neurobiological substrates of aggression may expand our search for targeted neuromodulation and pharmacological treatments.

## 1. Introduction

Violence is a pervasive issue worldwide, imposing a substantial burden on both communities and the lives of those affected (Mikton et al., 2016). At a population level, aggressive behaviors tend to manifest early in childhood and show a gradual decline throughout the developmental stages (Carroll et al., 2023). However, a significant portion of these children (between 6 and 11 %) may continue to exhibit elevated levels of aggression in late adolescence (Becht et al., 2016; Bongers et al., 2004; Fontaine et al., 2014) and in adulthood (Carlisi et al., 2020; Moffitt, 1993). In fact, aggressive behaviors are observed in various mental health problems in youths (e.g., Conduct Disorder, Oppositional Defiant Disorder, Disruptive Mood Dysregulation Disorder, Autism Spectrum Disorders) and adults (e.g., Antisocial Personality Disorder, Borderline Personality Disorder, and Intermittent Explosive Disorder, American Psychiatric Association, 2013). Identifying and managing aggression in childhood may have profound impact on reducing negative psychosocial outcomes and may improve the course of illnesses and well-being (Dodge et al., 2015).

Aggression is often defined as a behavior intended to harm others (Anderson & Bushman, 2002). However, it is an umbrella term that lumps together the idiosyncratic nature of aggression, particularly in regard to its motives and forms. For instance, reactive aggression (i.e., *impulsive, hostile, emotional, or defensive behaviors*, (Dodge & Coie, 1987; Wrangham, 2018) occurs in response to threats, provocation, or frustration and may produce relief from negative affect, which can be hedonically pleasant (Chester, 2017). Cross-species evidence has also highlighted a distinct type of aggressive behavior that occurs in the absence of provocation or threat, namely proactive aggression (i.e., *appetitive*, *premeditated, instrumental*, or *predatory behaviors* that are motivated by the obtainment of a goal or reward) (Dodge & Coie, 1987; Wrangham, 2018). From a psychometric perspective, previous meta-analysis indicated that reactive and proactive correlated substantially (*r* = 0.64), which is thought to be attributable to high item loadings on both factors (Polman et al., 2007). Nevertheless, factor analyses on the widely used Reactive-Proactive Questionnaire (RPQ) (Raine et al., 2006) support the two-factor structure of the functions of aggression (Cima et al., 2013; Raine et al., 2006; Toro et al., 2020). In turn, main forms in which aggression may occur include Physical Aggression (i.e., causing physical harm to others, e.g., hitting, biting, kicking, Buss & Durkee, 1957; Buss & Perry, 1992), Verbal Aggression (i.e., using speech to psychologically hurt others, e.g., shouting/screaming, insults, threats, Buss & Durkee, 1957; Buss & Perry, 1992), but also Indirect (i.e., *relational*, or *social* behaviors intended to harm social relationships, e.g., manipulation, damage relationships, Björkqvist, 2001; Crick & Grotpeter, 1995). These aggressive behaviors were originally assessed with the Buss-Durkee Hostility Inventory (Buss & Durkee, 1957), and the Buss-Perry Aggression Questionnaire (BPAQ)(Buss & Perry, 1992), more recently. The factor structure of the BPAQ is relatively stable across samples and languages, with moderate correlations (0.45 to 0.61) between its physical and verbal factors (Buss & Perry, 1992; Harris, 1997; Vigil-Colet et al., 2005). In the past decades, studies have attempted to disentangle the different forms and functions of aggression. However, the neurobiological mechanisms underpinning these types remains elusive.

In animals, the neurobiological circuitry involved in reactive aggression (aggressive behaviors in response to a threat) is relatively well characterized and includes the medial nucleus of the amygdala, ventromedial hypothalamus, and the periaqueductal grey (PAG) (Gregg & Siegel, 2001; Lischinsky & Lin, 2020; Panksepp & Zellner, 2004; Potegal & Nordman, 2023). In contrast, findings indicate that unprovoked aggression (e.g., quiet attack) also involves the amygdala (Block et al., 1980; Brutus et al., 1986; Egger & Flynn, 1963; Potegal & Nordman, 2023), but additionally recruit the lateral hypothalamus (Li et al., 2018; Panksepp, 1971; Potegal & Nordman, 2023; Shaikh et al., 1991; Smith & Flynn, 1980), septal area (Potegal & Nordman, 2023; Siegel & Skog, 1970), medial and lateral prefrontal cortex (PFC) (Proshansky et al., 1974; Siegel et al., 1975), and VTA (Piazza et al., 1986; Potegal & Nordman, 2023; Proshansky et al., 1974). In humans, reactive aggression appears to rely on similar brain structures than in rage attacks in animals (Blair, 2004; Blair, 2022) but is believed to be modulated by subregions of the prefrontal cortex, including the lateral orbitofrontal cortex (Blair, 2004) extending to the ventrolateral PFC/anterior insula (Bertsch et al., 2020; Blair, 2022; Dugré & Potvin, 2023a; Lickley et al., 2018; Sorella et al., 2021) and possibly the dorsolateral prefrontal cortex (Achterberg et al., 2020; Ibrahim et al., 2022). In contrast, proactive aggression (analogous to quiet-biting attacks) is thought to be characterized by activity in the amygdala, ventral striatum, medial orbitofrontal cortex, ventromedial PFC, and posterior cingulate cortex (Belfry & Kolla, 2021; Blair, 2022; Crowe & Blair, 2008; Romero-Martínez et al., 2022), which are commonly involved during reinforcement-based decision-making and motivational fMRI tasks (Dugré & Potvin, 2023b). Other meta-analytic findings suggest that the execution of retaliatory actions involved the activation of the dorsal striatum (caudate), left vlPFC, anterior insula, left postcentral gyrus, and dorsal anterior cingulate cortex (dACC) extending to the pre-SMA (Dugré & Potvin, 2023a). These motor outputs may thus distinguish physical from verbal behavioral forms of aggression.

Overall, our limited understanding of the neurobiological correlates of human aggression may be partially explained by an overreliance on animal models and a focus on predetermined brain regions rather than on whole-brain activation patterns. Moreover, previous meta-analyses on aggression varied in study inclusions due to their different objectives, leading to spatially distinct findings. For example, two meta-analyses included studies reporting participants with history of violence (e.g., psychiatric diagnosis or history of criminal violent behaviors). Wong et al. (2019) conducted meta-analysis across fMRI tasks and found a significant effect in the precuneus. Nikolic et al. (2022) conducted a meta-analysis specifically on anger/aggression-eliciting fMRI tasks and reported effects in the amygdala and middle temporal gyri. Finally, Raschle et al. (2015) conducted a meta-analysis on adolescents at risk for aggressive behaviors (disruptive behavior disorders) and found reduced activity in various regions including the dorsomedial prefrontal cortex, anterior insula, striatum, thalamus. However, these meta-analyses primarily focused on groups of individuals displaying aggressive behaviors (e.g., history of violence, disruptive behaviors disorders, aggression-prone), regardless of the severity. Consequently, it remains unknown whether the identified regions are linked to severity of aggression, hindering the understanding of its dimensional nature. Moreover, without relying on validated assessments of aggression and its subconstructs (e.g., in case-control studies), it is difficult to ascertain whether these findings may primarily explained by a particular motivation or form, and whether these sub-constructs rely on shared or distinct neural correlates.

The aim of the current manuscript was to thoroughly investigate the associations between brain activity and aggression through a coordinate-based meta-analysis of functional neuroimaging studies. More specifically, we examined the neural correlates of general aggression, its functions (i.e., reactive & proactive) and forms (i.e., physical & verbal). Based on previous findings, we hypothesized that aggression would be associated with subcortical, prefrontal cortices (medial and lateral) and temporal lobes (e.g., insula, temporal gyri). We also hypothesized that reactive and proactive aggression would show main differences in amygdala and striatum, respectively, while physical and verbal forms of aggression would be mainly associated with brain regions underpinning motor outputs (e.g., dorsal caudate, ACC, pre-SMA, (Dugré & Potvin, 2023a).

## 2. Methods

### 2.1. Eligibility criteria and study selection

#### 2.1.1. Literature search

The current meta-analysis of fMRI studies is derived from a larger systematic search also includes VBM studies on aggression (see Dugré & De Brito, 2024 for the meta-analysis of VBM studies). A systematic search strategy, using three search engines (Google Scholar, PubMed and Web of Science), was conducted up to May 1st 2023 to identify relevant studies. The following search terms were used: (aggress* OR violen* OR Predatory aggress* OR Instrumental aggress* OR Proactive aggress* OR Reactive agress* OR Impulsive aggress* OR Hostile aggress* OR Physical aggress* OR Verbal aggress*) AND (*neuroimaging* OR *fMRI* OR *VBM* OR *functional neuroimaging* OR *structural neuroimaging* OR *task-based* OR *voxel-based*). An additional search was conducted by cross-referencing the reference list of recent meta-analyses on task-based fMRI studies on aggression (Dugré & Potvin, 2023a; Nikolic et al., 2022; Wong et al., 2019). Irrelevant records and duplicates were first excluded. Full texts of the resulting studies were subsequently screened.

#### 2.1.2. Study selection

Articles were included if they met the following criteria: (1) original study published in a peer-reviewed journal; (2) inclusion of a validated measure of aggressive behaviors (i.e., questionnaires, interviews); (3) inclusion of a functional magnetic resonance imaging method; (4) conducted a group-comparison or a whole-brain regression assessing the dimensional relationship between voxels and the severity of aggression (5); reported findings from analyses across the whole-brain (i.e., voxelwise) [null or peak coordinates]. We excluded studies assessing aggressive behaviors within the scanner without a validated measure of aggression (e.g., severity of noise blast). These were excluded since it does not provide a validated way to compare severity of aggression between studies and the poor correlation with aggression questionnaire (see recently McCurry et al., 2024; Bertsch et al., 2022). See previous meta-analyses for neural correlates of laboratory-based aggression (Dugré & Potvin, 2023a; Puiu et al., 2020; Wong et al., 2019). In addition, we excluded studies reporting only one-sample *t*-test. This exclusion criterion was chosen given that one-sample t-test assess the main effect of the task and not inter-individual differences in aggression. Preferred Reporting Items for Systematic Reviews and Meta-Analyses (PRISMA) was followed across the meta-analysis steps.

### 2.2. Meta-analytic synthesis of fMRI studies

Coordinate-based meta-analytic approaches typically assess the spatial convergence of reported activation peaks (Eickhoff et al., 2012; Wager et al., 2009) or infer a meta-analytic effect size for grey matter voxel using effect sizes of reported peaks (Albajes-Eizagirre et al., 2019; Dugré et al., 2020). Given that variability in meta-analytic approaches may lead to similar yet distinct results (Albrecht et al., 2019; Enge et al., 2020), we sought to examine the reliability of neural correlates of aggression by overlapping threshold maps from both Activation Likelihood Estimation algorithm (Eickhoff et al., 2012) and Seed-based d Mapping (SDM-PSI, Albajes-Eizagirre et al., 2019) (see Supplementary Methods for detailed information about meta-analytic approaches). Given that our goal is to estimate the spatial convergence across neuroimaging studies, irrespectively of the task, we combined increased and decreased brain activity results to generate an aberrant brain activity map for each study. This approach was chosen due to the fact that the directionality of the effects (increased/decreased or positive/negative activity) depends on the tasks and contrasts between conditions.

In the SDM-PSI meta-analysis, variations in brain activity were identified using a more stringent threshold (*p* < 0.0001, 20 voxels) than usually recommended (*p* < 0.005, see Dugré et al., 2020; Radua et al., 2012) at a voxel-level, to avoid spurious results of the pooled effect size across increased and decreased brain activity coordinates. Residual heterogeneity of included studies was examined (I2 > 50 % indicates substantial heterogeneity) and potential publication bias was assessed via a meta-regression of the effect size by its standard error (Egger et al., 1997; Sterne et al., 2011). Subanalyses on the influence of studies reporting null findings and jackknife (relative contribution of each experiment) were also conducted. Moderators of main analyses such as sex, age group (i.e., <18 years old and ≥ 18 years old) and settings (i.e., community versus clinical/criminal samples) were tested using the Comprehensive Meta-Analysis software (Borenstein et al., 2020). As secondary analyses, whole-brain linear models were estimated with 50 random imputations with a more lenient statistical threshold of p < 0.005 uncorrected at a voxel-level and a cluster extent threshold of k > 10 voxels as used recently (see Dugré et al., 2020; Radua et al., 2012). These linear models were conducted to examine the degree of which effect sizes of brain activity was associated with sample’s severity of aggression (i.e., Percentage of Maximum Possible Score [POMP], see Dugré et al., 2020; Rogers & De Brito, 2016) and magnitude of case-control difference in severity of aggression (i.e., Hedge’s g).

In the ALE meta-analyses, since there is no effect size for any given voxel, variations in brain activity were identified using the usual recommended statistical threshold (*p* < 0.001 at a voxel-level, cFWE<0.05, Eickhoff et al., 2016). Specific ALE subanalyses were conducted by meta-analyzing peak coordinates of whole-brain regression studies assessing the dimensional relationship between aggression and whole-brain voxels. As an exploratory and descriptive analysis, we further explored whether some task domains may have contributed to the results. To do so, we manually annotated task contrasts, and examined the average *Z*-score across voxels of each brain regions identified in the main meta-analysis for each task domains. This was done only for general, reactive and proactive meta-analysis given the limited number of studies for physical and verbal. Only the most prevalent task domains were examined: Emotional Faces (e.g., fear, sad, happy), Negative Emotions (e.g., anger-scripts, passive viewing, anger induction), Cognition (e.g., stroop task, go/no-go, n-back), and Decision-Making (e.g., Monetary Incentive Delay Task, Colorado Balloon Game). Finally, we examined the robustness of our ALE findings against publication bias. Samartsidis et al. (2020) recently estimated roughly 30 unpublished studies with null effects for every 100 published ones. The fail-safe (FSN) metric (Rosenthal, 1979) evaluate the robustness of meta-analytic findings by calculating how many null studies need to be added to render effect sizes non-significant. In the context of ALE meta-analyses, adding a minimum of 30 % of added papers from the total sample before rendering null results should be inferred as robust. Since the ALE algorithm does not contain effect sizes, artificial studies were randomly generated from the original sample (e.g., number of peaks and sample size) and added to the original meta-analysis (see Acar et al., 2018); implementation by Enge et al., 2020 and Bortolini et al., 2024).

### 2.3. Associations with mental functions and neurotransmission systems

A meta-analytic co-activation modelling (MACM) approach was conducted to identify the potential mental functions associated with the aggression-related fMRI findings observed in the current study. To do so, we meta-analyzed studies from the Neuroquery repository (>21,083 experiments on healthy subjects) (Dockès et al., 2020) that reported at least one peak coordinate in a given Region-of-Interest (i.e., 8 mm sphere around center coordinates of our results). A meta-analysis (ALE) was then conducted to calculate spatial convergence across studies reporting a peak coordinate within the ROI (Eickhoff et al., 2012) implemented in NiMaRE (Salo et al., 2022). The resulting ALE meta-analytic map (z-map) would therefore reflect the general co-activation pattern of a given ROI across fMRI tasks.

Once the co-activation map was estimated, we calculated its spatial similarity with 13 data-driven task-based fMRI maps which summarize the last 20 years of research of meta-analyses of task-based neuroimaging studies (see https://neurovault.org/collections/13769/, (Dugré & Potvin, 2023b) as well as 19 receptor/transporter density maps (Hansen et al., 2022) which include serotonin (i.e., 5-HT_1A_, 5-HT_1B_, 5-HT_2A_, 5-HT_4_, 5-HT_6_, 5-HTT), dopamine (i.e., D_1_, D_2_, DAT), norepinephrine (i.e., NET), Histamine (i.e., H_3_), acetylcholine (i.e., α4β2, M_1_, VAChT), cannabinoid (i.e., CB_1_), opioid (i.e., MOR), glutamate (i.e., NMDA, mGluR_5_) and GABA (i.e., GABA_A/BZ_). Spatial associations with the tbfMRI maps and the PET density maps were conducted by correlating two sets of 226,654 voxels, via pearson’s correlation.

## 3. Results

### 3.1. Included studies in the neural bases of aggression

**Fig. 1.**
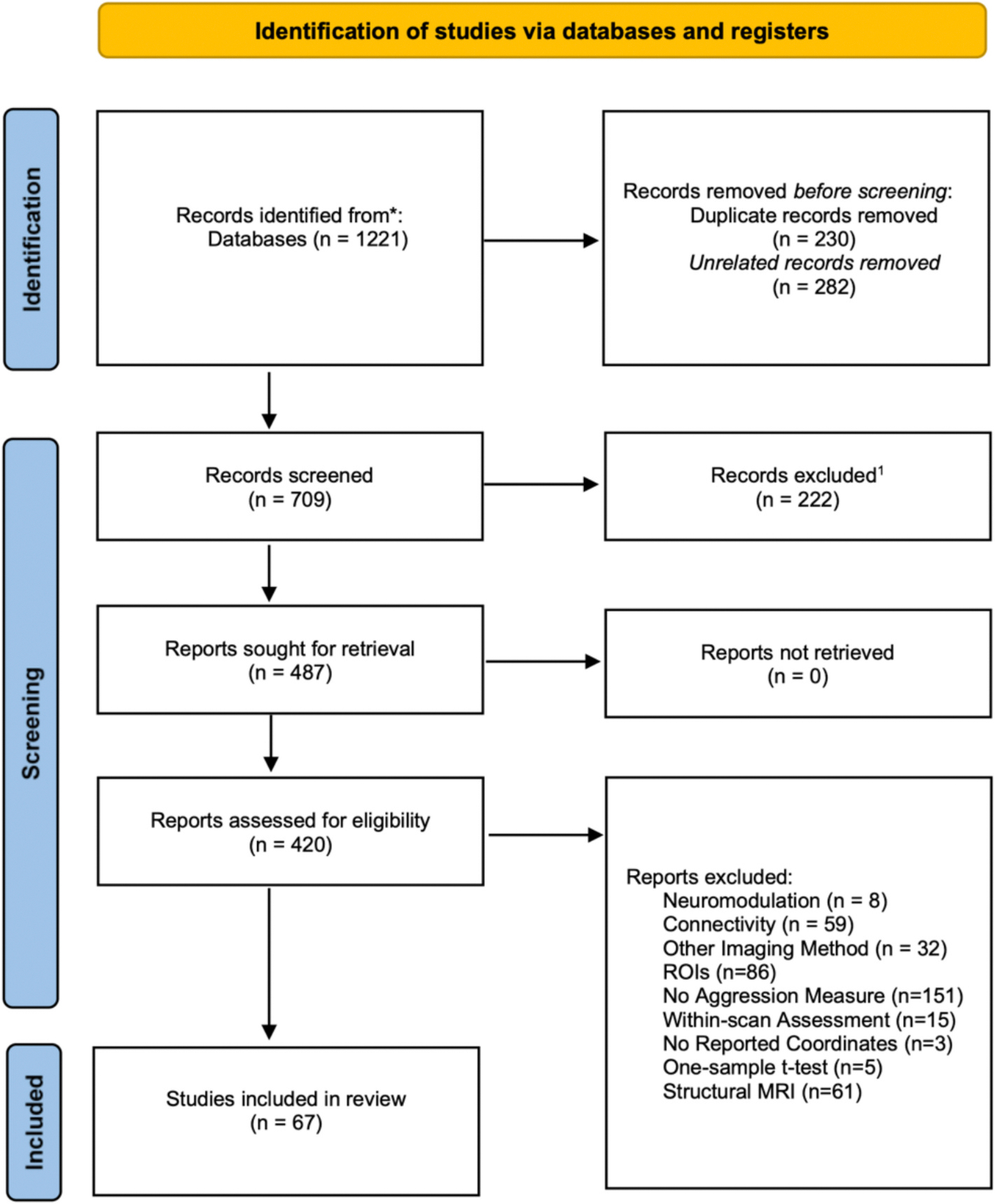
Flowchart representing the systematic literature search. ^1^ Reasons for exclusion were Literature Reviews (*n* = 138), Animal Studies (*n* = 19), Absence of fMRI or sMRI (*n* = 26), Case Studies (*n* = 6), Abstract, Book Chapter, and Thesis (*n* = 33).

The flowchart representing the literature search is displayed in Fig. 1. After screening, a total of 67 met the inclusion criteria for the current meta-analysis (see Table 1 for a Summary of the Included Studies). Across studies, 1767 cases were compared to 1621 controls, while dimensional analyses were conducted on 825 individuals. From the 67 studies, scores of validated measures of aggression were extracted for 76 independent samples and distributed based on their different functions (i.e., reactive-proactive) and forms (i.e., physical-verbal) of aggressive behaviors (see Table 1 for a description of the acronyms for each questionnaire). General Aggression scales include the CBCL-AGG, the LHA-AGG, RPQ, aggressive CD count, the FAF-AGG, and the Gunn-Robertson scale. Reactive Aggression scales include the BPAQ, RPQ-Reactive subscale, the FAI-Reactive subscale, the STAXI-AX-OUT, the K-FAF-Reactive subscale, the BDHI, the BAQ, and the BWAQ. Proactive Aggression scales include the RPQ-Proactive subscale, the FAI-Spontaneous subscale, TriPM-Meanness subscale, K-FAF-Spontaneous subscale, FAF-Spontaneous subscale, Illinois Bully Scale. Both Physical and Verbal Aggression were assessed via the BPAQ & BWAQ. Given the scarcity of fMRI studies on indirect aggression, we excluded this subconstruct (Li et al., 2020).

The unthresholded statistical maps of the SDM-PSI and ALE approaches are available (https://neurovault.org/collections/14865/).

### 3.2. Neural correlates of general aggression

#### 3.2.1. Main meta-analyses

Across the 102 experiments, the SDM-PSI method revealed variations in brain activity in subcortical (i.e., Centromedial Amygdala), frontal (i.e., ACC & ventrolateral PFC), temporal (i.e., Heschl’s gyrus, Inferior Temporal Gyrus & Temporal Gyrus), parietal (i.e., Inferior Parietal Lobule, Precuneus), and occipital (i.e., Visual Cortices) regions. The ALE algorithm showed spatial convergence across 653 foci in the left centromedial amygdala, the precuneus, right angular gyrus and the left intraparietal sulcus and middle temporal gyrus. Peak coordinates and results of the reliability analyses (i.e., null studies, jackknife) are presented in Supplementary Results.

**Fig. 2.**
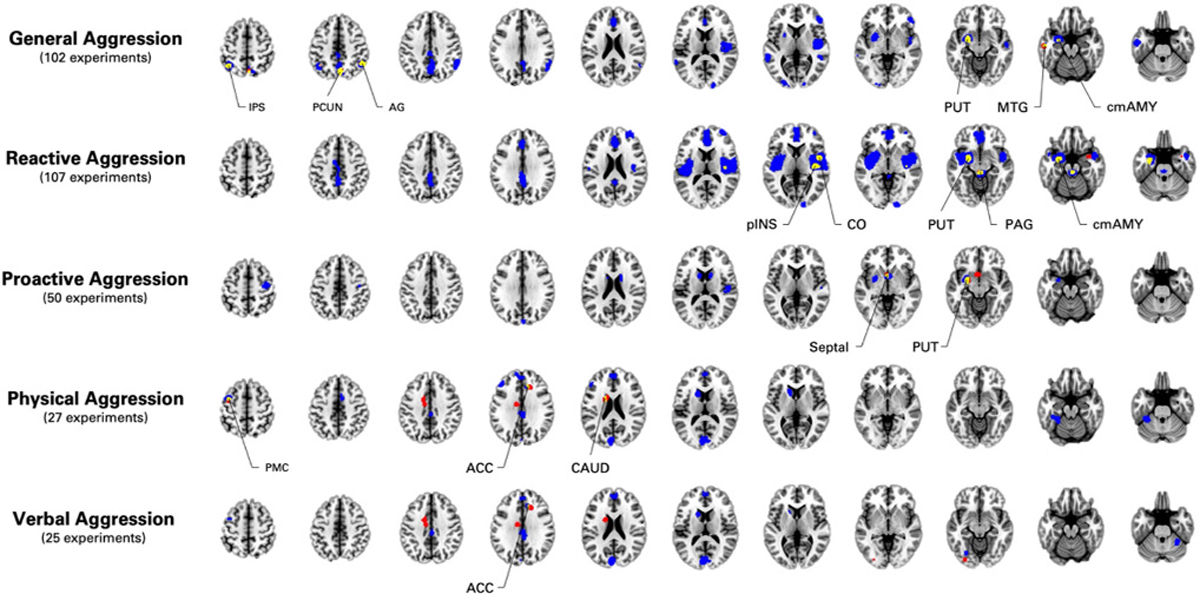
Summary of the Meta-Analytic Findings on Aggression. Blue Clusters = Activation Likelihood Estimation (ALE; p < 0.001, cmassFWE<0.05); Red Clusters = Seed-based d Mapping (SDM-PSI; *p* < 0.0001, 20 voxels). IPS = Intraparietal Sulcus; PCUN = Precuneus; AG = Angular Gyrus, PUT = Putamen; MTG = Middle Temporal Gyrus; cmAMY = Centromedial Amygdala; pINS = posterior Insula; CO = Central Opercular Cortex; PAG = Periaqueductal Grey; Septal = Basal Forebrain/Septal Area; PMC = Premotor Cortex; ACC = Anterior Cingulate Cortex; CAUD = Caudate Nucleus. (For interpretation of the references to colour in this figure legend, the reader is referred to the web version of this article.)

Spatial overlaps between findings from the two meta-analytic methods (Fig. 2, Table 2) were observed in the left centromedial amygdala (I^2^ = 32.18 %, Egger’s test *p* = 0.086), the precuneus (I^2^ = 17.61 %, Egger’s test *p* = 0.008), the left intraparietal sulcus (I^2^ = 8.35 %, Egger’s test *p* < 0.001), the right angular gyrus (I^2^ = 10.15 %, Egger’s test *p* = 0.004), and the middle temporal gyrus (I^2^ = 14.1 %, Egger’s test *p* = 0.005). Fail-safe analyses revealed that only the left amygdala (FSN = 132) and the precuneus (FSN = 66) were robust against publication bias, while the angular gyrus (FSN = 20), intraparietal sulcus (FSN = 11), middle temporal gyrus (FSN = 4) showed lower FSN than 30 % of added studies before rendering it non-significant.

Only the centromedial amygdala finding was positively related to the percentage of males per sample (Z = 2.32, *p* = 0.02). Subanalyses revealed no other significant relationships with moderators. General Aggression included 23 experiments for Emotional Faces, 30 experiments for Negative Stimuli, 17 experiments for Cognition, and 15 experiments on Decision-Making. Exploratory analyses revealed that the left amygdala (Z = 2.42, Z = 3.93), left intraparietal sulcus (Z = 3.01, Z = 2.94), the right angular gyrus (Z = 2.62, Z = 2.85), middle temporal gyrus (Z = 4.22, Z = 3.93) may have been driven by task involving emotional faces and/or negative stimuli, respectively (see Supplementary Fig. 1). However, effect of the precuneus appeared to be rather driven by cognitive tasks (Z = 4.02).

#### 3.2.2. Severity of general aggression

Dimensional studies exploring the link between aggression and voxels across the whole-brain (9 experiments, 65 foci) revealed no significant convergence of peak coordinates.

**Fig. 3.**
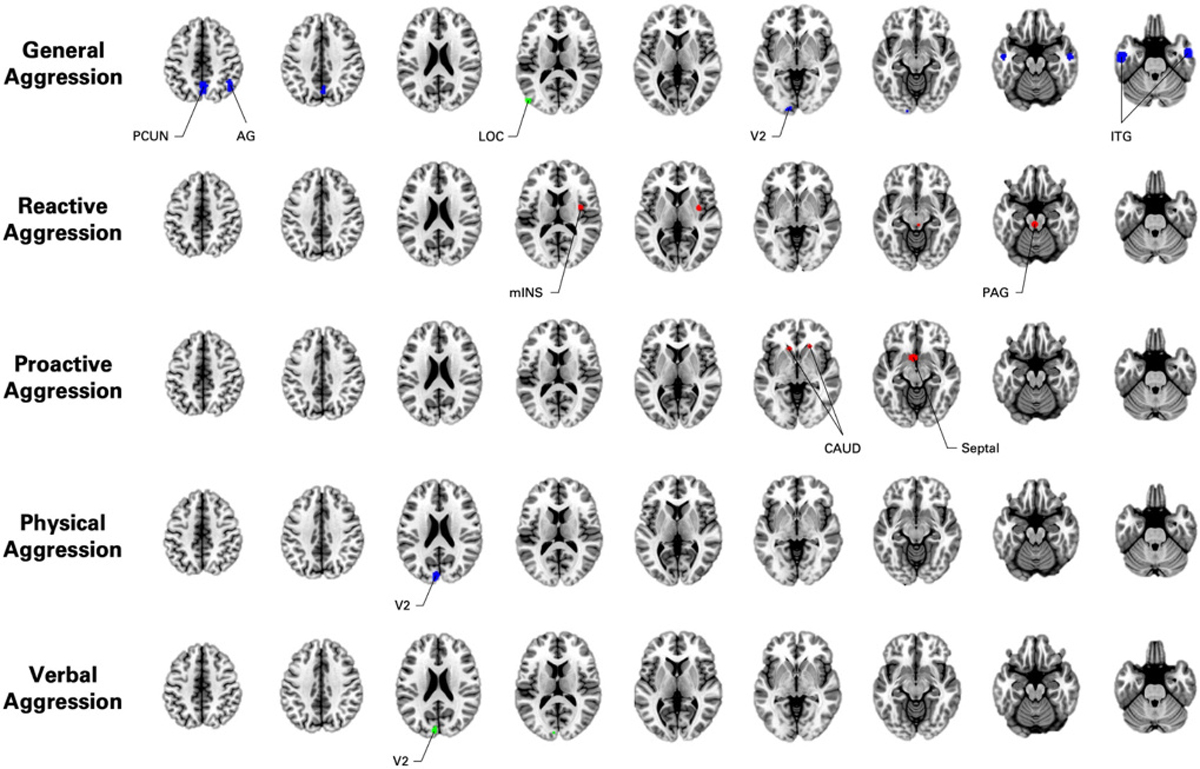
Summary of the Meta-Analytic Findings on Severity of Aggression. Blue Clusters = Percentage of Maximum Possible Score (POMP); Green Clusters = Effect size of Case-Control Difference (Hedges’ g); Red Clusters = Dimensional Studies. PCUN = Precuneus; AG = Angular Gyrus; LOC = Lateral Occipital Cortex; V2 = Secondary Visual Cortex; ITG = Inferior Temporal Gyrus; mINS = middle Insula; PAG = Periaqueductal Grey; CAUD = Caudate Nucleus; Septal = Septal Area. (For interpretation of the references to colour in this figure legend, the reader is referred to the web version of this article.)

**Fig. 4.**
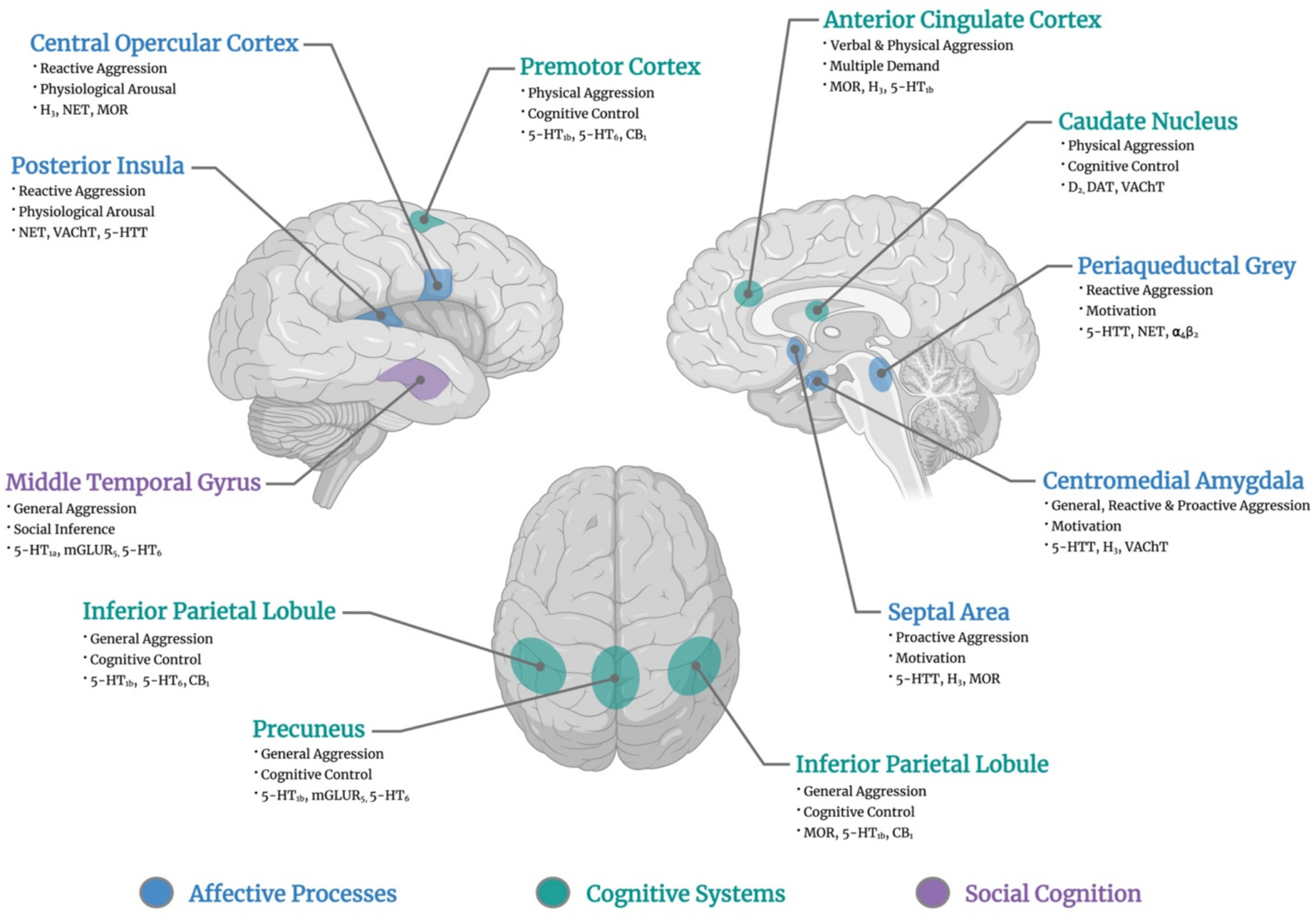
The Neural Bases of Aggression. Functional Characterization was conducted via spatial correlation between region-specific coactivation network (see supplementary material for more detailed information about the meta-analytic co-activation modelling) and 13 data-driven task-based co-activation networks (Dugré & Potvin, 2023b) and 19 PET/SPECT density maps (Hansen et al., 2022). Only the top correlated features are displayed. For the complete spatial similarity results, please refer to supplementary material for General Aggression (Tables S1–S2), Reactive Aggression (Tables S3–S4), Proactive Aggression (Tables S5–S6), Physical Aggression (S7–S8), and Verbal Aggression (S9-S10). This figure was created in BioRender.com (https://BioRender.com/i35b412).

The whole-brain meta-regression using samples’ severity of General Aggression (i.e., POMP score) revealed significant relationships with activity of the left inferior temporal cortex, right inferior temporal cortex, precuneus, secondary visual cortex, angular cortex, left Crus II, and premotor cortex (Table 3, Fig. 3, Supplementary Table 3 & Figs. 2–8). Effects found in these regions showed low heterogeneity (I^2^ < 5 %). All these regions showed greater relationships in clinical samples compared to community samples (Cochran’s Qs ranged from 4.3 to 13.24). Stronger relationships were observed in adult samples compared to youth samples in the precuneus (Q = 5.32, *p* = 0.021), Secondary Visual Cortex (Q = 5.73, *p* = 0.017) and the premotor cortex (Q = 4.62, *p* = 0.032). None of the results were significantly associated with percentage of males per sample.

The whole-brain meta-regression using Case-Control difference in severity of General Aggression (i.e., Hedges’ g) showed a positive association with the lateral occipital cortex (I^2^ < 5 %)(Table 3, Fig. 3, Supplementary Table 3 & Fig. 9), and was not significantly related to any moderators.

### 3.3. Neural correlates of reactive aggression

#### 3.3.1. Main meta-analyses

Across the 107 experiments, the SDM-PSI approach yielded variations in brain activity in the centromedial amygdala, the PAG, the posterior insula (pINS), anterior and posterior cingulate cortices, lateral PFC and the visual cortex. The ALE meta-analysis (560 foci) revealed spatial convergence in a cluster spanning the left centromedial nucleus and laterobasal amygdala, the right mid- and pINS and a cluster spanning the PAG to the pontine tegmentum (e.g., raphe nuclei). Peak coordinates and results of the reliability analyses (i.e., null studies, jackknife) are presented in Supplementary Results.

Spatial overlaps between findings from the two meta-analytic methods (Fig. 2, Table 2) were observed in the left centromedial amygdala (I^2^ = 6.82 %, Egger’s test *p* = 0.008), the pINS (I^2^ < 5 %, Egger’s test p < 0.001), the PAG (I^2^ < 5 %, Egger’s test p = 0.005), the central opercular cortex (I^2^ < 5 %, Egger’s test *p* = 0.001). Fail-safe analyses revealed that only the left amygdala (FSN = 187) and the pINS (FSN = 32) were robust against publication bias, while the central opercular cortex (FSN = 9) and the PAG (FSN = 4) showed lower FSN than 30 % of added studies before rendering it non-significant.

Assessing potential moderators showed greater effects in adults versus youths in the centromedial amygdala (Q = 4.34, *p* = 0.037). No other moderating effects were observed. Reactive Aggression included 25 experiments on Emotional Faces, 36 experiments for Negative Emotions, 14 experiments for Cognition and 11 experiments for Decision-Making. Exploratory analyses revealed that the left amygdala (Z = 3.48), central opercular cortex (Z = 2.95), and the PAG (Z = 3.14) may have been driven by task involving negative stimuli (see Supplementary Fig. 1). The effect found in the pINS, however, appear to be found in studies involving emotional faces (Z = 2.30), negative stimuli (Z = 2.78), and decision-making (Z = 2.39).

#### 3.3.2. Severity of reactive aggression

Dimensional studies (10 experiments, 48 foci) revealed spatial convergence in the right insula (mid-to-posterior) and a cluster spanning the PAG to the pontine tegmentum (Table 3, Fig. 3, Supplementary Table 3).

Whole-brain meta-regression using sample’s severity (i.e., POMP score) or Case-Control differences in Reactive Aggression (i.e., Hedges’ g) revealed no significant results.

### 3.4. Neural correlates of proactive aggression

#### 3.4.1. Main meta-analyses

Across the 50 experiments, the SDM-PSI approach revealed deficient activity in a cluster spanning the ventral putamen extending to the centromedial amygdala, the septal area, precentral gyrus, bilateral dorsal caudate, heschl’s gyrus and cuneus. The ALE algorithm (261 foci) only revealed spatial convergence in the left centromedial amygdala and the basal forebrain (septal area extending to the left nucleus accumbens). Peak coordinates and results of the reliability analyses (i.e., null studies, jackknife) are presented in Supplementary Results.

Spatial overlaps between findings from the two meta-analytic methods (Fig. 2, Table 2) were observed in the left centromedial amygdala (I^2^ = 11.26 %, Egger’s test *p* = 0.09), and the basal forebrain (I^2^ < 5 %, Egger’s test p = 0.008). Fail-safe analyses revealed that both the amygdala (FSN = 200) and the basal forebrain (FSN = 77) were robust against publication bias.

These findings were not significantly associated with any potential moderators (e.g., percentage of males, age group (i.e., youths versus adults), or setting (i.e., community versus clinical/criminal). Proactive Aggression included 8 experiments for Emotional Faces, 5 experiments for Negative Emotions, 6 experiments for Cognition, and 11 experiments for Decision-Making. Exploratory analyses revealed that the left amygdala was mainly driven by studies using negative stimuli (Z = 3.84), while the effect found in the basal forebrain appear distributed across decision-making (Z = 2.38), cognitive tasks (Z = 2.33) and emotional faces (Z = 2.06) (Supplementary Fig. 1).

#### 3.4.2. Severity of proactive aggression

Dimensional studies (3 experiments, 21 foci) revealed spatial convergence in the septal area and left and right caudate nucleus (Table 3, Fig. 3, Supplementary Table 3).

Whole-brain meta-regression using sample’s severity (i.e., POMP score) and Cases-Control differences (i.e., Hedges’ g) in Proactive Aggression revealed no significant relationships.

### 3.5. Neural correlates of physical aggression

#### 3.5.1. Main meta-analyses

Across the 27 experiments, the SDM-PSI approach showed variations in brain activity in several subcortical (i.e., Dorsal Caudate), frontal (i.e., dorsomedial & dorsolateral PFC, dorsal ACC, MCC, Premotor), temporal (i.e., Temporal Fusiform Cortex) and parietal and occipital regions (i.e., PCC, Visual Area 3). The ALE meta-analysis (167 foci) revealed deficient activity in the right dACC, left midcingulate cortex, left dorsal caudate, and the premotor motor cortex. Peak coordinates and results of the reliability analyses (i.e., null studies, jackknife) are presented in Supplementary Results.

Spatial overlaps between findings from the two meta-analytic methods (Fig. 2, Table 2) were observed in the Premotor Cortex (I^2^ = 13.83 %, Egger’s test *p* = 0.464), Dorsal Caudate (I^2^ = 19.83 %, Egger’s test *p* = 0.938), and the Dorsal ACC (I^2^ < 5 %, Egger’s test *p* = 0.552). Fail-safe analyses revealed that only the Dorsal Caudate (FSN = 26) was robust against publication bias, while the Dorsal ACC (FSN = 7) and the Premotor Cortex (FSN = 5) showed lower FSN than 30 % of added studies before rendering it non-significant.

Activity in these brain regions were not significantly associated with any potential moderators (e.g., percentage of males, age group (i.e., youths versus adults), or setting (i.e., community versus clinical/criminal).

#### 3.5.2. Severity of physical aggression

Dimensional studies (4 experiments, 17 foci) revealed no spatial convergence.

Whole-brain meta-regression using sample’s severity (i.e., POMP score) of Physical Aggression revealed significant relationship with the secondary visual cortex and the dACC (Table 3, Fig. 3, Supplementary Table 3 & Figs. 10–11). Effect found in the secondary visual cortex was significantly associated with percentage of males per sample (Z = 3.1, *p* < 0.0019) and adult samples (versus youths, Q = 12.68, p < 0.001). The dACC was related with greater males per sample (Z = 2.92, *p* < 0.0036), adults (Q = 10.43, *p* < 0.0012), and clinical samples (Q = 5.60, *p* < 0.018).

Whole-brain meta-regression using Case-Control differences in severity of Physical Aggression (i.e., Hedges’ g) showed no significant effect.

### 3.6. Neural correlates of verbal aggression

#### 3.6.1. Main meta-analysis

Across the 25 experiments, the SDM-PSI method showed significant effect in the visual cortex, dorsomedial PFC, dACC, premotor cortex, Caudate, Lobule VI, and occipital fusiform gyrus. The ALE meta-analysis (i.e., 152 foci) revealed deficient activity in the right dACC, left MCC, left dorsal caudate, and the extrastriate visual cortex (i.e., V4). Peak coordinates and results of the reliability analyses (i.e., null studies, jackknife) are presented in Supplementary Results.

Spatial overlap between findings from the two meta-analytic methods (Fig. 2, Table 2) was only in the dorsal ACC (I^2^ < 5 %, Egger’s test *p* = 0.982). Fail-safe analyses revealed it was not robust against publication bias (FSN = 6), showing lower FSN than 30 % of added studies before rendering it non-significant.

The dorsal ACC showed no significant relationship with any of the potential moderators.

#### 3.6.2. Severity of verbal aggression

Only one dimensional study was found (Li et al., 2020). The authors reported a significant effect in the primary auditory area.

Whole-brain meta-regression using sample’s severity (i.e., POMP score) of Verbal Aggression revealed no significant effect.

Whole-brain meta-regression using Case-Control difference was associated with activity of the visual cortex (i.e., Area 3) (Table 3, Fig. 3, Supplementary Table 3 & Fig. 12). Moderating analyses revealed significant relationship with percentage of males per sample (Z = 2.88, *p* = 0.0039). No other statistically significant effects were observed.

## 4. Discussion

The current study aimed to identify the brain circuits involved in human aggression. More precisely, we conducted coordinate-based meta-analyses of fMRI studies on the motivations (i.e., reactive and proactive aggression) and the forms (i.e., physical and verbal) in which aggressive behaviors typically occur. Our meta-analytic review revealed that general aggression was characterized by activity of the amygdala, precuneus, intraparietal sulcus, angular gyrus and the middle temporal gyrus. Moreover, while the amygdala was involved irrespectively of the motivation, reactive (i.e., pINS, PAG, central opercular cortex) and proactive (i.e., Basal Forebrain/Septal area) aggression were associated with distinct neural correlates. Similarly, physical aggression was related to activity of the premotor cortex, dorsal caudate and dACC, which was also found in verbal aggression. Our exploratory analyses aiming to further characterize the brain regions involved in human aggression revealed strong correspondence to affective (i.e., physiological arousal, motivation processes), cognitive (i.e., cognitive control, multiple-demand), and social cognition (i.e., social inference), as well as serotoninergic, dopaminergic, and cholinergic systems (see Fig. 4).

Animal research has previously highlighted, via different methods and samples, the importance of the medial amygdala, the (lateral & medial) hypothalamus, and the PAG in aggressive behaviors (Lischinsky & Lin, 2020; Panksepp & Zellner, 2004; Siegel & Victoroff, 2009). Across the scientific literature, there is a general agreement that the amygdala is involved in aggression, irrespective of the motivation (Haller, 2018; Siegel et al., 1999; Zhao et al., 2023), although some researchers posited that its nuclei might be associated with distinct motives (Haller, 2018). Nonetheless, the amygdala-medial hypothalamus-PAG circuit appears to be specific to threat response (i.e., reactive and/or defensive aggression) (Bertsch et al., 2020; Panksepp & Zellner, 2004). Our meta-analysis revealed that human reactive aggression involved the centromedial amygdala, the PAG as well as somatosensory areas (i.e., pINS, central opercular cortex) which are known to be associated with skin conductance responses to threatening stimuli (Taschereau-Dumouchel et al., 2020). These findings concur with our additional analyses showing that the brain circuit underpinning reactive aggression is mainly characterized by affective systems, including physiological arousal, as well as serotoninergic (i.e., SERT), and cholinergic systems (i.e., VAChT). In turn, proactive aggression was rather distinguished by the activity of the basal forebrain/septal area which partially overlapped with the nucleus accumbens. Early evidence suggests that the basal forebrain/septal area plays a major role in positive reinforcement learning (Olds & Milner, 1954) due to its central hub that connects the hippocampus to the lateral hypothalamus, ventral tegmental area and nucleus accumbens (Besnard & Leroy, 2022; Rizzi-Wise & Wang, 2021). Intriguingly, lesions to this particular region have been linked to a “septal rage”, a syndrome mainly characterized by frequent unprovoked attacks and viciousness by the lesioned rats (Albert & Chew, 1980), which may be facilitated via the projection from the septal area to the hypothalamus (Siegel & Skog, 1970; Wong et al., 2016). Finally, clinical work in humans has recently found a potential causal role of the hypothalamus in human aggressive behaviors (Benedetti-Isaac et al., 2015; Contreras Lopez et al., 2021; Gouveia et al., 2021; Gouveia et al., 2023; Micieli et al., 2017; Torres et al., 2020). The absence of meta-analytic findings in this small area should not cast doubt on its importance in aggression and may rather be partially explained by several factors including the coarse fMRI resolution, as well as the distortions due to its proximity to ventricules and blood vessels.

Across species, motor outputs such as the midbrain (i.e., PAG, VTA), and the dorsal striatum are necessary to produce a behavioral aggressive response (Lischinsky & Lin, 2020). The ACC may also play a non-negligible role in the aggressive response, potentially via a top-down regulation process of subcortical regions (i.e., amygdala, hypothalamus)(Jager et al., 2020; van Heukelum et al., 2021). Although these findings are mainly found in animal literature, the action of inflicting pain or removing points to an opponent in human include the left insula, the dorsal caudate, the primary somatosensory cortex, and the dorsal part of the ACC extending to the pre-supplementary motor area (Dugré & Potvin, 2023a). In line with these results, we showed that physical aggression was associated with the activity of the premotor cortex, the dorsal caudate and the dACC, while verbal aggression only showed significant activity in the latter. Structural and functional connectivity studies showed strong connections between the dACC, the dorsal striatum and the premotor cortex, via cortico-striatal loops (Alexander et al., 1986; Choi et al., 2012; Di Martino et al., 2008; Haber, 2016). The extensive projections from the dACC to the striatum (Haber, 2016) (Kolling et al., 2016) suggests that the former may be implicated in the selection of an appropriate action (Rudebeck et al., 2008) and may enable activity of brain regions involved in motor planning and execution such as the dorsal caudate and premotor cortex (Haber, 2016; Kolling et al., 2016).

## 5. Limitations

Several limitations need to be acknowledged. First, image-based meta-analyses are usually preferred over coordinate-based meta-analyses, although it is often difficult to access unthresholded images from original studies (Salimi-Khorshidi et al., 2009). Image-based meta-analysis may provide a more precise estimates of the neural correlates of aggression. Second, several results in this meta-analysis showed potential publication bias. While it is not uncommon in neuroimaging meta-analyses (Jennings & Van Horn, 2012), our meta-analysis spanning across different tasks may have exacerbate publication bias in particular regions. Despite that our approach allow to examine the neural correlates of aggression across multiple brain processes, the heterogeneity of tasks and neurocognitive domains impact the base rate of brain regions which may have explained why many regions were not robust against publication bias. For instance, regions recruited by many neurocognitive domains may be more likely to robust to fail-safe analyses. Follow up neuroimaging meta-analysis on aggression may clarify the robustness of our findings across different task domains. Third, we manually annotated the validated measures according to the presence of items related to general aggression and to the motives and forms. However, the structure of aggression may not be clearly delineated by its motivational aspects and forms (as presented in this study) and may include other types of aggression (e.g., relational aggression or alcohol-driven aggression) (Chester et al., 2023). For example, retaliatory behaviors can occur days or even weeks after the provocation, when the negative affect is no longer present. This specific type of aggression, labeled delayed aggression (Chester, 2024), may therefore be characterized as more predatory than hostile-impulsive behaviors. This suggests that the timing of the aggression may be a crucial component in distinguishing between different types of aggressive behaviors. Fourth, despite that the different approaches were carried out to explore the linear relationship between the severity of aggression and local brain activity (i.e., dimensional studies, POMP score, Hedges’ g), biases in literature may exist in the choice of statistical approaches (i.e., dimensional/case-control) in community and clinical samples. Indeed, most studies use a case-control analytic approach, which may obscure the dimensional nature of aggression. Fifth, the use of a group-based approach may have introduced statistical artefacts in our findings. For instance, overlap in groups (experiments) between reactive, proactive and general aggression may have explained similarities in the involvement of the amygdala, while overlap in physical and verbal experiments may have explained the activity of the dACC. Group overlaps were relatively small for general-reactive (29.9 %), general-proactive (28.3 %), reactive-proactive (34.3 %), suggesting potentially distinct groups contributing to the amygdala. However, very high overlap was found for physical and verbal aggression (100 %) suggesting that the dACC may have been driven by the same studies. Although this region may be involved in both forms of aggression, interpretation of this results should be made with caution. Finally, we performed a meta-analysis of neuroimaging studies irrespective of the specific fMRI tasks used. This approach was chosen to identify the main convergent brain regions across the literature while addressing the challenges posed by the wide range of tasks and contrasts in these studies. Indeed, since the directionality of effects depends on the tasks and contrasts between conditions, the activity of brain regions reported in our meta-analysis cannot be interpreted as increased/decreased or positive/negative.

## 6. Conclusion

In summary, our meta-analysis revealed that aggression was significantly associated with brain activity several regions already identified as core hubs of aggression via animal studies. More specifically, general aggression was mainly characterized by activity of the amygdala, dorsal parietal regions and middle temporal gyrus, which underscored the interaction between motivation, cognitive control and social cognition in our understanding of aggression. We also found that motivation (i.e., reactive and proactive) was distinguished by subcortical regions and somatosensory regions, while the forms (i.e., physical and verbal) were rather linked to the dACC, and motor outputs. Our findings are consistent with lesion studies in human highlighting the role of frontal, temporal and amygdala regions in aggression including murder, physical assault, and rape (Darby et al., 2018; Dugré & Potvin, 2022). These may be crucial targets for personalized treatments including neuromodulation techniques and pharmacological interventions.

## Supplementary Material

1

## Figures and Tables

**Table 1:** Description of the Included Studies on Aggression.

First Author, Date	Samples				Controls (n=)	Analysis	Aggression Measures	fMRI Task
	Group Description	n	Mean Age	Males (%)				

(Aggensteiner et al., 2022)	Oppositional Defiant Disorder/Conduct Disorder	108	13.2	82.4 %	69	Case Control	CBCL-AGG; RPQ-Total; RPQ-Reactive; RPQ-Proactive	Emotional Faces
(Aghajani et al., 2021)	Conduct Disorder with Limited Prosocial Emotions	19	16.4	100.0 %	31	Case Control	RPQ-Total; RPQ-Proactive; RPQ-Reactive	Emotional Recognition & Emotional Resonance
(Beames et al., 2020)	Healthy Subjects	21	21.0	57.1 %	24	Both	BPAQ-Total	Unsolvable Anagram
(Bertsch et al., 2018)	Borderline Personality Disorder	30	26.9	0.0 %	28	Case Control	STAXI AX-OUT	Approach Avoidance Task (Faces)
(Bertsch et al., 2022)	Borderline Personality Disorder	48	29.6	0.0 %	28	Case-Control	BPAQ-Total	Social Threat Aggression Paradigm
(Blair et al., 2021)	Adolescents from Residential Care Facility	98	16.0	69.4 %	–	Dimension	Aggression Incident Reports	The Looming Task
(Bobes et al., 2013)	Violent Men in Community	25	30.6	100.0 %	29	Case Control	RPQ-Total; RPQ-Proactive; RPQ-Reactive; BDHI-Total	Fearful Faces
(Bubenzer-Busch et al., 2016)	Attention-Deficit/Hyperactivity Disorder & Disruptive Behavior Disorders	27	10.9	100.0 %	27	Both	CBCL-AGG; BPAQ-Total; BPAQ-Physical; BPAQ-Verbal; RPQ-Total; RPQ-Reactive; RPQ-Proactive	mPSAG
(Coccaro et al., 2007)	Intermittent Explosive Disorder	10	34.3	50.0 %	10	Case-Control	LHA-AGG; BPAQ-Total	Emotional Faces
(Coccaro et al., 2022)	Intermittent Explosive Disorder	19	35.0	42.0 %	26	Case Control	LHA-AGG; BPAQ-AGG	V-SEIP task
(Coccaro et al., 2021)	Healthy Subjects	26	32.0	50.0 %	–	Dimension	LHA-AGG	V-SEIP task
(Cohn et al., 2013)	Disruptive Behavior Disorders (Desisters)	25	17.6	80.0 %	26	Case Control	RPQ-Total	Fear Conditioning Task
	Disruptive Behavior Disorders (Persisters)	25	17.3	72.0 %	–			
(Crowley et al., 2010)	Adolescents with Antisocial Substance Disorder	20	16.5	100.0 %	20	Case-Control	Peak Aggressive Behavior Rating Scale	Colorado Balloon Game
(Crowley et al., 2015)	Males with Antisocial Substance Disorder	20	16.5	100.0 %	20	Case-Control	Peak Aggressive Behavior Rating Scale	Decision-Making Behavioral Task
	Females with Antisocial Substance Disorder	21	16.2	0.0 %	20			
(Cremers et al., 2016)	Intermittent Explosive Disorder	17	32.5	58.8 %	14	Case Control	LHA-AGG	Emotional Faces
(Crum et al., 2023)	Adolescents with Substance Misuse & Rule-Breaking Problems	55	16.7	60.0 %	125	Case Control	CBCL-AGG	Passive Avoidance Task
(Decety et al., 2009)	Conduct Disorder	8	16 to 18	NA	8	Case-Control	Aggressive CD Symptom Count	Empathy for Pain
(Eijsker et al., 2019)	Patients with Misophonia	22	33.2	27.0 %	21	Case-Control	BPAQ-Total; BPAQ-Physical; BPAQ-Verbal	Stop Signal Task
(Fairchild et al., 2014)	Conduct Disorder	20	17.0	0.0 %	20	Case Control	Aggressive CD Symptom Count	Emotional Faces
(Gan et al., 2016)	Clinical & Sub-Clinical Intermittent Explosive Disorder	9	34.4	100.0 %	9	Case Control	STAXI2-AX-OUT	PSAP
(Garciá-Martí et al., 2013)	Treatment Resistant Schizophrenia with Auditory Hallucination	32	NA	NA	–	Dimension	BPRS-Hostility	Emotional Words (Auditory)
(Gatzke-Kopp et al., 2009)	Externalizing Disorders	19	13.6	100.0 %	11	Case-Control	CBCL-AGG	MIDT
(Gregory et al., 2015)	Antisocial Personality Disorder (without Psychopathy)	20	36.8	100.0 %	18	Case Control	RPQ-Total; RPQ-Reactive;	Probabilistic Response Reversal Task
	Antisocial Personality Disorder (with Psychopathy)	12	40.1	100.0 %	–		RPQ-Proactive	
(Hazlett et al., 2012)	Borderline Personality Disorder	33	31.6	39.0 %	32	Case-Control	BPAQ-Total	Repeated Emotional Pictures
	Schizotypal Personality Disorder	28	35.9	57.0 %	–			
(Heesink et al., 2017)	Veterans with Anger Problems	27	36.4	100.0 %	30	Case Control	BPAQ-Physical; BPAQ-Verbal	Fear-and-Escape Task (FAET)
(Heesink et al., 2018)	Veterans with Anger Problems	30	36.3	100.0 %	29	Case Control	BPAQ-Physical; BPAQ-Verbal	Affective Stimuli (IAPS)
(Herpertz et al., 2008)	Childhood-Onset Conduct Disorder	22	14.7	100.0 %	22	Case Control	CBCL-AGG	Affective Stimuli (IAPS)
	Attention-Deficit/Hyperactivity Disorder	13	14.0	100.0 %	13			
(Herpertz et al., 2017)	Borderline Personality Disorder	33	26.2	0.0 %	30	Case-Control	BPAQ-Total	Anger-Aggression Scripts
	Borderline Personality Disorder	23	30.7	100.0 %	26			
(Ibrahim et al., 2019)	Autism Spectrum Disorder/Disruptive Behavior Disorders	18	12.7	88.9 %	19	Case-Control	CBCL-AGG	Emotional Faces
(Jakobi et al., 2022)	Attention-Deficit/Hyperactivity Disorder	78	34.2	43.6 %	78	Both	RPQ-Proactive; RPQ-Reactive	Emotional Faces
(Jiang et al., 2018)	Healthy Controls	19	20.0	52.6 %	20	Case Control	BPAQ-Total	TAP
(Kesner et al., 2020)	Medical Students (High Xenophobic)	19	23.0	52.6 %	19	Case Control	BPAQ-Total; BPAQ-Physical; BPAQ-Verbal	Affective Stimuli (Refugees/Terrorists)
(Kim et al., 2018)	Delinquent Adolescents	8	14.5	75.0 %	17	Case-Control	CBCL-AGG	Rest
(Konzok et al., 2021)	Healthy Subjects (High Externalizing Traits)	31	23.1	50.8 %	30	Case Control	TriPM-Meanness	ScanSTRESS
(Konzok et al., 2022)	Healthy Subjects (High Externalizing Traits)	32	23.6	50.0 %	31	Case Control	TriPM-Meanness, K-FAF-Spontaneous; K-FAF-Reactive; BPAQ-Physical; BPAQ-Verbal; RPQ-Proactive; RPQ-Reactive	mTAP
(Krauch et al., 2018)	Borderline Personality Disorder	20	16.4	0.0 %	20	Case Control	BPAQ-Total	Anger-Aggression Scripts
	Borderline Personality Disorder	34	25.7	0.0 %	32			
(Kumari et al., 2006)	Violent Patients with Schizophrenia	12	34.0	100.0 %	13	Case-Control	Gunn-Robertson Scale	N-Back Task
	Antisocial Personality Disorder	10	31.3	100.0 %	13			
(Kumari et al., 2009)	Violent Patients with Schizophrenia	13	34.5	100.0 %	13	Case Control	Gunn-Robertson Scale	Fear Elicitation (Shock)
	Antisocial Personality Disorder	13	32.9	100.0	14			
(Li et al., 2020)	Primary School Students	77	10.2	45.5 %	–	Dimension	BWAQ-Total; BWAQ-Physical; BWAQ-Verbal	Rest
(Martinelli et al., 2021)	Healthy Subjects	50	15.6	50.0 %	–	Dimension	BPAQ-Physical	Emotional Faces
(Mathur et al., 2023)	Residential Treatment Program	42	16.2	61.0 %	41	Case-Control	RPQ-Total^9^; RPQ-Reactive^9^; RPQ-Proactive^9^	Retaliation Task
(McCloskey et al., 2016)	Intermittent Explosive Disorder	20	33.2	60.0 %	20	Case-Control	LHA-AGG	Emotional Faces
(Michalska et al., 2016)	Child from Mental Health and Pediatric Clinics	107	10.1	48.0 %	–	Dimension	RPQ-Reactive	Harm scenarios
(Moeller et al., 2014)	Intermittent Explosive Disorder	11	33.5	100.0 %	38	Case-Control	STAXI AX-OUT	Stroop Task
(Murray et al., 2023)	Youths from Low Income Families	128	15.9	42.0 %	–	Dimension	CBCL-AGG	MIDT
(Passamonti et al., 2010)	Early-Onset Conduct Disorder	11	17.7	100.0 %	18	Case Control	Aggressive CD Symptom Count	Emotional Faces
	Adolescence-Onset Conduct Disorder	11	17.1	100.0 %	–			
(Pawliczek, Derntl, Kellermann, Gur, et al., 2013)	Students (High Trait Aggression)	21	22.2	100.0 %	18	Case-Control	BPAQ-Total; BPAQ-Physical; BPAQ-Verbal; FAI-Total; FAI-Spontaneous; FAI-Reactive	Unsolvable Anagrams
(Pawliczek, Derntl, Kellermann, Kohn, et al., 2013)	University Students (High Externalizing Traits)	17	22.2	100.0 %	16	Case Control	BPAQ-Total; BPAQ-Physical; BPAQ-Verbal	Stop Signal (Affective Faces)
(Perino et al., 2019)	Adolescents (with School or Legal intervention)	24	16.2	50.0 %	–	Dimension	University of Illinois Bully Scale	The Catch Game
(Prehn, Schulze, et al., 2013)	Borderline Personality Disorder/Antisocial Personality Disorder	15	27.9	100.0 %	17	Case Control	FAF-Aggression; FAF-Spontaneous; FAF-Reactive	N-Back (w/wo affective stimuli)
(Prehn, Schlagenhauf, et al., 2013)	Emotionally Hyporeactive Offenders	11	27.6	100.0 %	13	Case-Control	FAF-Spontaneous; FAF-Reactive	Monetary Decision-Making Task
	Emotionally Hyperreactive Offenders	12	27.8	100.0 %	–			
(Rahrig et al., 2021)	Active Coping Group	11	35.1	45.0 %	9	Case Control	BAQ	TAP
(Repple et al., 2018)	Healthy Controls	22	24.8	100.0 %	20	Case Control	BPAQ-Total; BPAQ-Physical; BPAQ-Verbal	mTAP
(Schroder et al., 2019)	Patients with Misophonia	21	33.1	28.0 %	23	Case-Control	BPAQ-Total; BPAQ-Physical; BPAQ-Verbal	Clip Viewing
(Seok & Cheong, 2020)	Intermittent Explosive Disorder	15	28.5	100.0 %	15	Both	LHA-AGG; BPAQ-Total	Affective Videos
(Sethi et al., 2018)	Primary Psychopathy (Community)	50	19.8	82.0 %	82	Case-Control	BPAQ-Total	Match-To-Sample (Faces)
	Secondary Psychopathy (Community)	100	19.5	58.0 %	–			
(Shao & Lee, 2017)	University Students (High Psychopathic Traits)	29	20.4	48.3 %	23	Case-Control	PPI-SCI	Face Familiarity
(Soloff et al., 2017)	Borderline Personality Disorder	31	30.0	0.0 %	–	Dimension	LHA-AGG	Go/No-Go Task
(Szycik et al., 2017)	Violent Video Game Users	15	22.8	100.0 %	15	Case Control	K-FAF-AGG	Socio-Affective Situations
(Taubner et al., 2021)	Violent Offenders	25	19.9	100.0 %	–	Dimension	RPQ-Total	Interactive Video
	Healthy Controls	21	20.0	100.0	–			
(Tonnaer et al., 2017)	Violent Offenders	16	35.8	100.0 %	18	Case-Control	RPQ-Total; RPQ-Reactive; RPQ-Proactive; BPAQ-Total	Emotional Stories
(Vanova et al., 2022)	Healthy Subjects (University Network)	22	24.1	31.8 %	–	Dimension	TriPM-Meanness	Lexical Decision Task
(Weidler et al., 2019)	OPRM genotype (G-)	39	25.2	100.0 %	20	Case Control	BPAQ-Total; BPAQ-Physical; BPAQ-Verbal; RPQ-Proactive; RPQ-Reactive	TAP
(White et al., 2016)	Disruptive Behavior Disorders	30	15.0	63.3 %	26	Case Control	RPQ-Proactive^9^; RPQ-Reactive^9^	Social Fairness Game
(White et al., 2018)	Disruptive Behavior Disorders	31	14.6	71.0 %	27	Case-Control	RPQ-Reactive^9^	The Looming Task
(Yoder et al., 2015)	Healthy Subjects who Watch Mixed Martial Arts	43	25.0	100.0 %	–	Dimension	PPI-SCI	Passive Viewing MMA
(Zhang et al., 2023)	Conduct Disorder	101	15.9	64.4 %	77	Case-Control	RPQ-Total; RPQ-Reactive; RPQ-Proactive	Passive Avoidance Task

Note. CBCL-AGG = Child Behavior Checklist – Aggression Syndrome Scale (Achenbach & Rescorla, 2000); RPQ = Reactive & Proactive Aggression(Dodge & Coie, 1987; Raine et al., 2006); BPAQ = Buss-Perry Aggression Questionnaire (Buss & Perry, 1992); STAXI-AX-OUT = State-Trait Anger Expression Inventory - Anger Expression OUT (Spielberger et al., 1999); LHA = Life History of Aggression (Coccaro et al., 1997); BWAQ = Buss-Warren Aggression Questionnaire (Buss & Warren, 2000); TriPM = Triarchic Psychopathy Measure (Drislane et al., 2014); K-FAF = Short version of the Factors of Aggression Questionnaire (Heubrock & Petermann, 2008); BDHI = Buss-Durkee Hostility Inventory (Buss & Durkee, 1957); BAQ = Brief Aggression Questionnaire (Webster et al., 2015); FAF/FAI = Factors of Aggression Questionnaire (Hampel & Selg, 1975); Peak Aggressive Behavior Rating Scale (Crowley et al., 2001); BPRS-Hostility = Brief Psychiatric Rating Scale (Overall & Gorham, 1988); Gunn-Robertson Violence Scale (Gunn & Robertson, 1976); University of Illinois Bully Scale (Espelage & Holt, 2001); PPI-SCI = Psychopathic Personality Inventory-Self-Centered Impulsivity (Lilienfeld & Andrews, 1996).

**Table 2:** Converging neurobiological substrates of Aggression across SDM-PSI and ALE methods.

Results	MNI Coordinates			Peak Intensity		I2 Statistics (%)	Overlapping voxels
	x	y	z	ALE-Z	SDM-Z		

**GENERAL AGGRESSION**							
Centromedial Amygdala	−25	−3	−12	5.11	5.78	28.14	162
Precuneus	3	−66	47	4.34	6.04	15.51	110
Intraparietal Sulcus (IPL)	−38	−54	52	4.24	6.44	8.35	79
Angular Gyrus (IPL)	49	−49	44	4.34	5.79	7.85	59
Middle Temporal Gyrus	−55	−15	−21	4.10	5.56	13.45	36
**REACTIVE AGGRESSION**							
Centromedial Amygdala	−27	−2	−16	3.75	9.98	5.18	252
Posterior Insula	38	−18	13	3.13	9.85	<1.0	120
Periaqueductal Grey	0	−30	−16	3.51	7.49	<1.0	80
Central Opercular Cortex	47	−1	4	4.22	7.64	<1.0	55
**PROACTIVE AGGRESSION**							
Basal Forebrain/Septal Area	−2	8	−8	5.16	3.68	37.26	12
Centromedial Amygdala	−24	−2	−12	5.05	5.93	19.11	11
**PHYSICAL AGGRESSION**							
Premotor Cortex	−38	2	57	3.93	6.88	9.74	55
Caudate Nucleus	−16	4	20	4.23	5.88	19.83	24
Anterior Cingulate Cortex	13	28	31	3.36	5.99	2.03	15
**VERBAL AGGRESSION**							
Anterior Cingulate Cortex	12	28	31	3.27	6.45	<1.0	12

Note. SDM-PSI results were thresholded using p < 0.0001 uncorrected, 20 voxels. ALE results were thresholded using p < 0.001 uncorrected, cmassFWE < 0.05.

**Table 3:** SDM-PSI and ALE Meta-analytic Results on Severity of Aggression.

Results	MNI Coordinates			Peak Intensity (Z-score)	Cluster size (Voxels)
	x	y	z		

**GENERAL AGGRESSION**					
*SDM-PSI - Severity of Aggression*					
Inferior Temporal Gyrus	−52	−14	−24	4.06	190
Precuneus	4	−54	52	3.51	206
Inferior Temporal Gyrus	54	−10	−24	3.65	180
Secondary Visual Cortex	−14	−98	−8	3.43	48
Angular Gyrus	42	−56	46	3.04	47
Crus II	−32	−72	−42	2.85	17
Premotor Cortex	−38	6	50	2.99	13
*SDM-PSI - Case-Control Difference*					
Lateral Occipital Gyrus	−42	−82	14	3.45	48
*ALE - Dimensional Studies*					
None					
**REACTIVE AGGRESSION**					
*SDM-PSI - Severity of Aggression*					
None					
*SDM-PSI - Case-Control Difference*					
None					
*ALE - Dimensional Studies*					
Mid-Insula	36	−4	10	4.53	53
Periaqueductal Grey	0	−30	−18	4.05	52
**PROACTIVE AGGRESSION**					
*SDM-PSI - Severity of Aggression*					
None					
*SDM-PSI - Case-Control Difference*					
None					
*ALE - Dimensional Studies*					
Basal Forebrain/Septal Area (ext. to the NAcc)	−6	8	−10	4.61	64
Caudate	−12	22	0	4.30	19
Caudate	20	26	0	4.30	19
**PHYSICAL AGGRESSION**					
*SDM-PSI - Severity of Aggression*					
Secondary Visual Cortex	−4	−84	22	3.01	60
Dorsal Anterior Cingulate Cortex	14	26	32	2.95	10
*SDM-PSI - Case-Control Difference*					
None					
*ALE - Dimensional Studies*					
None					
**VERBAL AGGRESSION**					
*SDM-PSI - Severity of Aggression*					
None					
*SDM-PSI - Case-Control Difference*					
Secondary Visual Cortex	−6	−90	20	2.72	14

Note. SDM-PSI results were thresholded using *p* < 0.005 uncorrected, 10 voxels. ALE results were thresholded using p < 0.001 uncorrected, cmassFWE < 0.05.

## Data Availability

Data will be made available on request.

## Appendix A. Supplementary data

Supplementary data to this article can be found online at https://doi.org/10.1016/j.avb.2025.102035.
