# Supplementary material for: The dark sides of the brain: A systematic review and meta-analysis of functional neuroimaging studies on trait aggression
Source: Aggress Violent Behav. Author manuscript; Available in PMC 2026 May 29. (PMC13218644; doi:10.1016/j.avb.2025.102035)
Supplement: 1 [file NIHMS2170716-supplement-1.pdf]

– SYSTEMATIC REVIEW & META-ANALYSIS –

The Dark Side of the Brain:

A Systematic Review and Meta-Analysis of Functional Neuroimaging Studies on Trait Aggression

Jules R. Dugré, PhD [1]; Christian Hopfer, PhD [2]; Drew E. Winters, PhD [2]

[1] School of Psychology and Centre for Human Brain Health, University of Birmingham, Birmingham, UK

[2] Department of Psychiatry, University of Colorado School of Medicine, Anschutz Medical Campus, CO, USA

Corresponding authors

Jules Roger Dugré, PhD
School of Psychology and Centre for Human Brain Health, University of Birmingham, Birmingham, UK; B15 2TT;
Email: jules.dugre@gmail.com
&
Drew Winters, PhD
Department of Psychiatry, Anschutz Medical Campus, Colorado, USA; CO 80045
Email: Drew.winters@cuanschutz.edu

Table of Contents

1. Supplementary Method #1: Meta-analytic Procedure *3*

2. Supplementary Method #2: Meta-analytic Approaches *3*

2.1. Spatial convergence approach (i.e., Activation Likelihood Estimation, ALE) **3**

2.2. Effect-size approach Seed-based d Mapping with Permutation of subject images, SDM-PSI) **4**

2.3. Meta-analytic Co-Activation Modelling **4**

3. Supplementary Method #2: Psychometric Scales assessing Aggression *4*

4. Supplementary Results: Sample Description, NULL findings & Jackknife Analyses *5*

4.1. General Aggression **5**

4.2. Reactive Aggression **5**

4.3. Proactive Aggression **6**

4.4. Physical Aggression **6**

4.5. Verbal Aggression **7**

5. Supplementary Tables *8*

Supplementary Table 1. Converging neurobiological substrates of Aggression across SDM-PSI and ALE methods **8**

Supplementary Table 2. SDM-PSI and ALE Meta-analytic Results on Aggression **9**

Supplementary Table 3. SDM-PSI and ALE Meta-analytic Results on Severity of Aggression **11**

Supplementary Table 4 ..... **12**

Supplementary Table 5 ..... **13**

Supplementary Table 6 ..... **14**

Supplementary Table 7 ..... **15**

Supplementary Table 8 ..... **16**

Supplementary Table 9 ..... **17**

Supplementary Table 10 ..... **18**

Supplementary Table 11. ..... **19**

Supplementary Table 12 ..... **20**

Supplementary Table 13 ..... **21**

6. Supplementary Figures *22*

Supplementary Figure 1. ..... **22**

Supplementary Figure 2. ..... **23**

Supplementary Figure 3. ..... **24**

Supplementary Figure 4. ..... **25**

Supplementary Figure 5. ..... **26**

Supplementary Figure 6. ..... **27**

Supplementary Figure 7. ..... **28**

Supplementary Figure 8. ..... **29**

Supplementary Figure 9. ..... **30**

Supplementary Figure 10. ..... **31**

Supplementary Figure 11. ..... **32**

Supplementary Figure 12. ..... **33**

1. Supplementary Method #1: Meta-analytic Procedure

For each study, coordinates reported in Talairach coordinates were converted into MNI (Montreal Neurologic Institute) space and effect sizes were extracted and converted into a t-value before running the meta-analysis. Reliability of meta-analytic results was defined by spatial overlap between results of the ALE and the SDM-PSI meta-analytic methods. Analyses were conducted irrespective of the directionality of the effect (hyper- & hypoactivation) given that directionality depend on the task and group contrasts and due to the poor reporting of estimates to adequately assess whether results are decreased activation or actual deactivation. Finally, studies reporting null coordinates were compared to those with a significant effect on the rate of youth samples, clinical/criminal samples, males per sample, the method of analysis, sample's severity of aggression, and case-control difference in severity of aggression.

In the SDM-PSI meta-analysis, aberrant brain activity was examined using a stringent threshold at a voxel-level ($p<0.0001$, 20 voxels threshold) to avoid spurious results. Residual heterogeneity of included studies was examined ($I2>50$ % indicates substantial heterogeneity) and potential publication bias was assessed via a meta-regression of the effect size by its standard error (55, 56). Subanalyses on the influence of studies reporting null findings and jackknife (relative contribution of each experiment) were also conducted. Moderators of main analyses such as sex, age group (i.e., <18 years old and ≥18 years old) and settings (i.e., community versus clinical/criminal samples) were tested using the Comprehensive Meta-Analysis software (57). As secondary analyses, whole-brain linear models were estimated with 50 random imputations with a more lenient statistical threshold of $p<0.005$ uncorrected at a voxel-level and a cluster extent threshold of $k > 10$ voxels as used recently (52) which optimally balance sensitivity and specificity (58). These linear models were conducted to examine the degree of which effect sizes of brain activity was associated with sample's severity of aggression (i.e., POMP score), and case-control difference in severity of aggression (i.e., Hedge's g).

In the ALE meta-analyses, aberrant brain activity was examined using a recommend statistical threshold ($p<0.001$ at a voxel-level, cFWE<0.05, (59). Specific ALE subanalyses were conducted by meta-analyzing peak coordinates of studies assessing the dimensional relationship between aggression and whole-brain voxels.

2. Supplementary Method #2: Meta-analytic Approaches

2.1. <u>Spatial convergence approach (i.e., Activation Likelihood Estimation, ALE)</u>

Experiments' coordinates were used for spatial convergence using the Activation Likelihood Estimate method (GingerALE version 3.0.2,[1, 2]; http://www.brainmap.org/ale/). For each experiment, a gaussian probability distribution was modelled around each coordinate foci, weighted by the number of subjects in the experiment. This is done to account for spatial uncertainty due to template and between-subject variance. Also, it ensures that multiple coordinates from a single experiment does not jointly influence the modeled activation value of a single voxel. The probabilities of all activation foci in an experiment were then combined to form a modeled activation map. Voxel-wise ALE scores were computed from the union across all these experiment maps. The size of the supra-threshold clusters is then compared against a null distribution of cluster sizes derived from simulation of datasets.

2.2. Effect-size approach Seed-based d Mapping with Permutation of subject images, SDM-PSI)

The current voxel-wise meta-analysis was performed using the Seed-based d Mapping with Permutation of Subject Images (SDM-PSI version 6.11) [3, 4]. Briefly, the SDM-PSI is a voxel-based meta-analysis software using peak coordinates and their t-values as reported from the original studies, to impute, for each study, multiple effect-size maps (Hedges' effect size) of contrast results (increased and decreased activations). Maps are then combined in a standard random-effects model considering sample size, intra-study variability and between-study heterogeneity [5], and multiple imputations are pooled using Rubin's rules [3]. The familywise error rate (FWER) of the results is calculated using a subject-based permutation test. SDM-PSI uses MetaNSUE [6] to estimate the maximum likely effect size within the lower and upper bounds of possible effects sizes for each study separately and then adds realistic noise.

2.3. Meta-analytic Co-Activation Modelling

Meta-analytic Co-Activation Modelling (MACM) was conducted to identify the co-activation pattern of each resulting cluster in healthy subjects. Briefly, we meta-analyzed studies from the BrainMap repository that reported at least one peak coordinate in a given Region-of-Interest (i.e., 12mm sphere around center coordinates). The BrainMap environment include a repository of neuroimaging studies which contain brain coordinates and metadata (e.g., sample size, behavioral categories) for more than 21,083 experiments. An activation likelihood estimation (ALE) algorithm was then used to calculate spatial convergence across studies [1] implemented in NiMaRE [7]. The resulting ALE meta-analytic map (z-map) therefore reflect the co-activation pattern of a given ROI across task.

Once the co-activation map has been estimated, we further calculated its spatial similarity with 13 data-driven task-based (tb)fMRI maps[8] (see https://neurovault.org/collections/13769/)and 19 receptor/transporter density maps [9] which include serotonin (i.e., 5-HT_{1A} , 5-HT_{1B} , 5-HT_{2A} , 5-HT_4 , 5-HT_6 , 5-HTT), dopamine (i.e., D_1, D_2 , DAT), norepinephrine (i.e., NET), Histamine (i.e., H_3), acetylcholine (i.e., α4β2, M_1 , VAChT), cannabinoid (i.e., CB_1), opioid (i.e., MOR), glutamate (i.e., NMDA, $mGluR_5$) and GABA (i.e., $GABA_{A/BZ}$). Spatial associations with the tbfMRI maps and the PET density maps were conducted by correlating two sets of 226,654 voxels.

3. Supplementary Method #2: Psychometric Scales assessing Aggression

Across studies, a wide range of psychometric measures were used to assess aggression including the Brief Aggression Questionnaire (BAQ) [10], Buss-Durkee Hostility Inventory (BDHI) [11], Buss-Perry Aggression Questionnaire (BPAQ) [12], Buss-Warren Aggression Questionaire [13], Child Behavior Checklist – Aggression Syndrome Scale (CBCL-AGG) [14], Reactive & Proactive Aggression Questionnaire (RPQ) [15, 16], State-Trait Anger Expression Inventory - Anger Expression Out (STAXI-AX-OUT)[17], Life History of Aggression (LHA) [18], Triarchic Psychopathy Measure (TriPM) [19], Factors of Aggression Questionnaire (FAF)[20] and its short version (K-FAF) [21], Peak Aggressive Behavior Rating Scale (PABR) [22], Hostility items of the Brief Psychiatric Rating Scale (H-BPRS) [23], Gunn-Robertson Violence Scale [24], University of Illinois Bully Scale [25], and Psychopathic Personality Inventory-Self-Centered Impulsivity (PPI-SC) [26]. These measures were then classified as belonging to General Aggression (i.e., across motivation [proactive & reactive] and forms [physical & verbal]), Proactive (i.e., across forms [physical & verbal]), Reactive (i.e.,

across forms [physical & verbal]), Physical (i.e., across motivation [proactive & reactive]), and Verbal (i.e., across motivation [proactive & reactive]).

General Aggression scales include the CBCL-AGG, the LHA-AGG, RPQ, aggressive CD count, the FAF-AGG, and the Gunn-Robertson scale. Reactive Aggression scales include the BPAQ, RPQ-Reactive subscale, the FAI-Reactive subscale, the STAXI-AX-OUT, the K-FAF-Reactive subscale, the BDHI, the BAQ, and the BWAQ. Proactive Aggression scales include the RPQ-Proactive subscale, the FAI-Spontaneous subscale, TriPM-Meanness subscale, K-FAF-Spontaneous subscale, FAF-Spontaneous subscale, Illinois Bully Scale. Both Physical and Verbal Aggression were assessed via the BPAQ & BWAQ Physical and Verbal subscales.

4. Supplementary Results: Sample Description, NULL findings & Jackknife Analyses

4.1. General Aggression

A total of 35 peer-reviewed studies were included in the meta-analysis on General Aggression, comprising 42 independent samples and a total of 102 experiments. About half of these samples were youths (51.2%) and most samples were recruited in clinical/criminal settings (80.49%). Mean age was 23.11 (SD=8.55) and average rate of males per sample was 72.58% (SD=31.4%). Average severity of aggression was 54.00% (SD=21.83%), and the average Case-Control Difference in severity of General Aggression, calculated via Hedges' g, was 2.67 (SD=1.51).

A total of 102 experiments were included in the meta-analysis, with 13 experiments reporting null results. Experiments with significant results significantly differ from those with null findings regarding the number of clinical samples (χ^2=26.05, p<0.001), the method of analysis (χ^2=4.35, p=0.037), and sample's severity of aggression (Mann Whitney U=347.00, p=0.028). Including these 13 experiments in the ALE meta-analysis did not alter the replicability of the findings.

Jackknife analyses revealed low potential that the centromedial amygdala (28 contributing experiments, Average per experiment=3.54%, Maximum contribution=18.54%), and the precuneus (19 contributing experiments, Average per experiment=5.22%, Maximum contribution=17.7%) was driven by a small number of studies. However, a smaller number of experiments explained most of the ALE score in the angular gyrus (14 contributing experiments, Average per experiment=7.09%, Maximum contribution=26.8%), the intraparietal sulcus (11 contributing experiments, Average per experiment=9.02%, Maximum contribution=28.9%), and the middle temporal gyrus (12 contributing experiments, Average per experiment=8.27%, Maximum contribution=27.6%)

4.2. Reactive Aggression

A total of 39 published studies reported a measure of Reactive Aggression, which comprised 42 independent samples and a total of 107 experiments. Only 21.4% of samples included youths, and more than half included participants from a clinical/criminal setting (64.3%). Mean age was 24.64 (SD=8.28) and average rate of males per sample was 59.63% (SD=36.86%). Average severity of

aggression was 51.57% (SD=13.03%), and the average Case-Control Difference in Reactive Aggression, was 1.69 (SD=1.03).

A total of 107 experiments were included in the meta-analysis, with 15 experiments reporting null results. The latter experiments only differed from those reporting significant effects in terms of the rates of youth samples (χ^2=4.78, p=0.029). Including these 15 experiments in the ALE meta-analysis did not alter the replicability of the findings.

Jackknife analyses revealed a low potential that the centromedial amygdala (39 contributing experiments, Average per experiment=2.54%, Maximum contribution=11.01%), and the Posterior Insula (25 contributing experiments, Average per experiment=3.972%, Maximum contribution=18.99%) was driven by a small number of studies. However, a smaller number of experiments explained most of the ALE score in the Temporal Pole (21 contributing experiments, Average per experiment=4.73%, Maximum contribution=31.6%), Central Opercular Cortex (18 contributing experiments, Average per experiment=5.52%, Maximum contribution=31.4%), and the periaqueductal gray (16 contributing experiments, Average per experiment=6.21%, Maximum contribution=29.33%)

4.3. Proactive Aggression

A total of 19 published studies reported a measure of Proactive Aggression, which comprised 21 independent samples with a total of 50 experiments. About one-third of samples included youths (33.3%), and more than half recruited participants in a clinical/criminal setting (61.9%). Mean age was 24.79 (SD=8.30) and average rate of males per sample was 58.86% (SD=36.96%). Average severity of aggression was 35.54% (SD=19.49%), and Case-Control Difference in measure of Proactive Aggression, calculated via Hedges' g, was 1.89 (SD=1.21).

A total of 50 experiments were included in the meta-analysis, with 10 experiments reporting null findings. The latter experiments significantly differed from those with significant effects regarding the rates of youth samples (χ^2=3.95, p=0.047), clinical samples (χ^2=5.43, p=0.020), sample's severity of proactive aggression (Mann-Whitney U=109.0, p=0.027), and case-control difference in severity of aggression (Mann-Whitney U=62.0, p=0.001). Including these 10 experiments did not alter the replicability of the findings.

Jackknife analyses revealed that the Basal Forebrain (13 contributing experiments, Average per experiment=7.65%, Maximum contribution=27.7%), and the centromedial amygdala (9 contributing experiments, Average per experiment=11.05%, Maximum contribution=26.2%) may have been driven by a small number of studies.

4.4. Physical Aggression

A total of 11 published studies reported a measure of Physical Aggression, which comprised 12 independent samples with a total of 27 experiments (302 cases, 294 controls). Only 16.7% of samples included youths, and less than half recruited participants in a clinical/criminal setting

(41.7%). Mean age was 21.76 (SD=8.44) and average rate of males per sample was 64.3% (SD=34.7%). Average severity of aggression was 46.64% (SD=13.69%), and Case-Control Difference in measure of Physical Aggression, calculated via Hedges' g, was 1.57 (SD=.96).

A total of 27 experiments were included in the meta-analysis, with 10 experiments reporting null findings. Experiments with null findings only differed from those reporting significant effects on the rates of youth samples (χ^2=4.54, p=0.033). Including these 10 experiments in the ALE meta-analysis did not alter the replicability of the findings.

Jackknife analyses revealed a moderate potential that the Dorsal Caudate (8 contributing experiments, Average per experiment=12.45%, Maximum contribution=30.88%) was driven by a small number of studies. However, results in Midcingulate cortex (8 contributing experiments, Average per experiment=12.44%, Maximum contribution=39.9%), the Dorsal Anterior Cingulate Cortex (9 contributing experiments, Average per experiment=11.07%, Maximum contribution=42.6%), and Premotor Cortex (3 contributing experiments, Average per experiment=33.2%, Maximum contribution=35.6%) show high potential that the ALE score may have been driven by a small number of studies.

4.5. Verbal Aggression

A total of 10 published studies reported a measure of Verbal Aggression, which comprised 11 independent samples with a total of 25 experiments. Only 9.1% of samples included youths, and less than half recruited participants in a clinical/criminal setting (45.5%). Mean age was 22.25 (SD=8.67) and average rate of males per sample was 61.01% (SD=34.4%). Average severity of aggression was 56.6% (SD=7.99%), and Case-Control Difference in measure of Verbal Aggression, calculated via Hedges' g, was .88 (SD=.60).

A total of 25 experiments were included in the meta-analysis, with 10 experiments reporting null findings. Experiments with null findings did not differ from those reporting significant effects on any of between-study variables. Including these 10 experiments in the ALE meta-analysis did not alter the replicability of the findings.

Jackknife analyses revealed a moderate to high potential that the right dACC (7 contributing experiments, Average per experiment=14.23%, Maximum contribution=40.03%), the left MCC (8 contributing experiments, Average per experiment=12.45%, Maximum contribution=41.81%), the left caudate (6 contributing experiments, Average per experiment=16.6%, Maximum contribution=33.9%) and the extrastriate visual cortex (3 contributing experiments, Average per experiment=33.22%, Maximum contribution=49.81%) were driven by a small number of studies.

5. Supplementary Tables

Supplementary Table 1. Converging neurobiological substrates of Aggression across SDM-PSI and ALE methods

Results	MNI Coordinates			Peak Intensity		I2 Statistics (%)	Overlapping voxels
	x	y	z	ALE-Z	SDM-Z		
**GENERAL AGGRESSION**							
Centromedial Amygdala	-25	-3	-12	5.11	5.78	28.14	162
Precuneus	3	-66	47	4.34	6.04	15.51	110
Intraparietal Sulcus (IPL)	-38	-54	52	4.24	6.44	8.35	79
Angular Gyrus (IPL)	49	-49	44	4.34	5.79	7.85	59
Middle Temporal Gyrus	-55	-15	-21	4.10	5.56	13.45	36
**REACTIVE AGGRESSION**							
Centromedial Amygdala	-27	-2	-16	3.75	9.98	5.18	252
Posterior Insula	38	-18	13	3.13	9.85	< 1.0	120
Periaqueductal Grey	0	-30	-16	3.51	7.49	< 1.0	80
Central Opercular Cortex	47	-1	4	4.22	7.64	< 1.0	55
**PROACTIVE AGGRESSION**							
Basal Forebrain	-2	8	-8	5.16	3.68	37.26	12
Centromedial Amygdala	-24	-2	-12	5.05	5.93	19.11	11
**PHYSICAL AGGRESSION**							
Premotor Cortex	-38	2	57	3.93	6.88	9.74	55
Caudate Nucleus	-16	4	20	4.23	5.88	19.83	24
Anterior Cingulate Cortex	13	28	31	3.36	5.99	2.03	15
**VERBAL AGGRESSION**							
Anterior Cingulate Cortex	12	28	31	3.27	6.45	< 1.0	12

Note. SDM-PSI results were thresholded using $p<0.0001$ uncorrected, 20 voxels. ALE results were thresholded using $p<0.001$ uncorrected, cmassFWE<0.05.

Supplementary Table 2. SDM-PSI and ALE Meta-analytic Results on Aggression

Results	MNI Coordinates			Peak Intensity (Z-score)	Cluster size (Voxels)
	x	y	z		
**GENERAL AGGRESSION**					
SDM-PSI meta-analysis					
Precuneus	2	-56	38	7.27	647
Centromedial Amygdala	-22	0	-16	7.25	500
Heschl's Gyrus	46	-18	8	6.89	514
Inferior Parietal Lobule	56	-46	38	6.89	287
Intraparietal Sulcus	-36	-60	50	7.12	208
Ventral Anterior Cingulate Cortex	-2	36	22	6.68	152
Ventrolateral PFC	46	40	6	6.68	151
Inferior Temporal Gyrus	-52	-10	-28	6.44	140
Posterior Middle Temporal Gyrus	-58	-42	4	6.22	118
Lateral Occipital Cortex (Area 4)	-26	-90	6	6.09	73
Primary Visual Area	16	-96	10	6.62	52
Middle Temporal Gyrus	56	-14	-12	5.80	26
Posterior Middle Temporal Gyrus	52	-42	6	5.90	23
ALE meta-analysis					
Centromedial Amygdala	-24	-4	-12	5.31	115
Precuneus	4	-66	48	4.61	103
Angular Gyrus	50	-50	44	4.48	52
Intraparietal Sulcus	-38	-54	50	4.39	53
Middle Temporal Gyrus	-56	-16	-20	4.43	44
**REACTIVE AGGRESSION**					
SDM-PSI meta-analysis					
Centromedial Amygdala	-30	-4	-12	10.62	2814
Posterior Insula	38	-12	6	10.51	2652
Ventral Anterior Cingulate Cortex	0	38	16	7.55	1349
Posterior Cingulate Cortex	-2	-44	40	6.84	498
Periaqueductal Grey	0	-32	-16	8.32	323
Lateral PFC	36	54	16	6.89	199
Primary Visual Area	16	-96	0	6.77	96
ALE meta-analysis					
Centromedial Amygdala	-24	-2	-12	5.22	221
Posterior Insula	38	-18	4	4.1	100
Temporal Pole	36	2	-22	4.56	39
Central Opercular Cortex	48	-2	4	4.17	44
Periaqueductal Grey	0	-30	-20	3.91	79
**PROACTIVE AGGRESSION**					
SDM-PSI meta-analysis					
Putamen (extending to cmAMY)	-26	0	-8	7.28	219
Precentral Gyrus	42	-12	50	6.36	151
Basal Forebrain	2	8	-4	6.85	94
Heschl's Gyrus	48	-22	10	6.13	65
Dorsal Caudate	14	2	20	6.37	44
Dorsal Caudate	-12	8	12	6.02	36
Cuneus	2	-86	28	6.28	28
ALE meta-analysis					
Basal Forebrain	-2	8	-8	5.16	95

Centromedial Amygdala	-24	-2	-12	5.05	90
**PHYSICAL AGGRESSION**					
SDM-PSI meta-analysis					
Visual Cortex (V3)	-8	-84	18	7.30	309
Temporal Fusiform Cortex	-34	-38	-22	7.13	206
Dorsomedial PFC	4	48	24	6.55	186
Dorsal Caudate	-16	12	10	7.16	157
Posterior Cingulate Cortex	2	-34	30	6.24	123
Premotor Cortex	-38	4	58	7.47	112
Dorsolateral PFC	-42	34	28	7.17	85
Midcingulate Cortex	8	4	44	6.67	31
Dorsal Anterior Cingulate Cortex	12	26	32	6.33	24
ALE meta-analysis					
Midcingulate Cortex	-12	-6	34	3.88	124
Dorsal Caudate	-16	2	20	4.42	97
Dorsal Anterior Cingulate Cortex	16	26	30	4.16	55
Premotor Cortex	-38	0	56	4.21	55
**VERBAL AGGRESSION**					
SDM-PSI meta-analysis					
Visual Cortex (V3)	-6	-82	24	7.12	376
Dorsomedial PFC	4	48	24	6.60	156
Posterior Cingulate Cortex	6	-30	30	6.32	155
Lobule VI	30	-52	-26	6.20	62
Caudate	-16	14	8	6.25	47
Dorsal Anterior Cingulate Cortex	12	28	32	6.50	31
Premotor Cortex	-38	2	58	5.99	27
Occipital Fusiform Gyrus	-28	-72	-12	6.30	21
ALE meta-analysis					
Midcingulate Cortex	-12	-6	34	3.99	149
Dorsal Anterior Cingulate Cortex	16	26	32	4.28	62
Dorsal Caudate	-18	2	20	4.10	60
Visual Cortex (V4)	-30	-84	-8	4.52	33

Note. SDM-PSI results were thresholded using $p<0.0001$ uncorrected, 20 voxels. ALE results were thresholded using $p<0.001$ uncorrected, cmassFWE<0.05.

Supplementary Table 3. SDM-PSI and ALE Meta-analytic Results on Severity of Aggression

Results	MNI Coordinates			Peak Intensity	Cluster size
	x	y	z	(Z-score)	(Voxels)
**GENERAL AGGRESSION**					
SDM-PSI - Severity of Aggression					
Inferior Temporal Gyrus	-52	-14	-24	4.06	190
Precuneus	4	-54	52	3.51	206
Inferior Temporal Gyrus	54	-10	-24	3.65	180
Secondary Visual Cortex	-14	-98	-8	3.43	48
Angular Gyrus	42	-56	46	3.04	47
Crus II	-32	-72	-42	2.85	17
Premotor Cortex	-38	6	50	2.99	13
SDM-PSI - Case-Control Difference					
Lateral Occipital Gyrus	-42	-82	14	3.45	48
ALE - Dimensional Studies					
None					
**REACTIVE AGGRESSION**					
SDM-PSI - Severity of Aggression					
None					
SDM-PSI - Case-Control Difference					
None					
ALE - Dimensional Studies					
Mid-Insula	36	-4	10	4.53	53
Periaqueductal Grey	0	-30	-18	4.05	52
**PROACTIVE AGGRESSION**					
SDM-PSI - Severity of Aggression					
None					
SDM-PSI - Case-Control Difference					
None					
ALE - Dimensional Studies					
Basal Forebrain (ext. to the NAcc)	-6	8	-10	4.61	64
Caudate	-12	22	0	4.30	19
Caudate	20	26	0	4.30	19
**PHYSICAL AGGRESSION**					
SDM-PSI - Severity of Aggression					
Secondary Visual Cortex	-4	-84	22	3.01	60
Dorsal Anterior Cingulate Cortex	14	26	32	2.95	10
SDM-PSI - Case-Control Difference					
None					
ALE - Dimensional Studies					
None					
**VERBAL AGGRESSION**					
SDM-PSI - Severity of Aggression					
None					
SDM-PSI - Case-Control Difference					
Secondary Visual Cortex	-6	-90	20	2.72	14

Note. SDM-PSI results were thresholded using p<0.005 uncorrected, 10 voxels. ALE results were thresholded using p<0.001 uncorrected, cmassFWE<0.05.

Supplementary Table 4. (General Aggression) Spatial Similarity between Region-specific co-activation network (MACM) and Mental Processes

	Region-of-Interest				
	Centromedial Amygdala	Precuneus	Intraparietal Sulcus	Angular Gyrus	Middle Temporal Gyrus
Action	0.261	0.395	0.493	0.396	-0.058
Auditory Perception	0.211	0.053	0.138	0.200	0.108
Cognitive Control	0.208	0.676	0.840	0.742	0.047
Face Detection	0.495	0.145	0.265	0.167	0.126
Language	0.213	0.305	0.480	0.401	0.327
Motivation	0.624	0.224	0.226	0.247	0.137
Multi Demand	0.254	0.472	0.596	0.628	0.092
Physiological Arousal	0.427	0.237	0.287	0.417	0.000
Social Inference	0.104	0.038	-0.046	-0.035	0.635
Social Representation	0.234	0.101	0.124	0.113	0.601
Spatial Attention	-0.062	0.346	0.250	0.226	-0.024
Spatial Memory	0.096	0.264	0.163	0.140	0.024
Valuation	0.136	0.094	0.051	0.083	0.278

Note. Spatial similarity was established using Pearson's correlation across 226,654 voxels. Statistical maps of mental processes derived from Dugré et Potvin (BioRxiv) and are available (https://neurovault.org/collections/13769/).

Supplementary Table 5. (General Aggression) Spatial Similarity between Region-specific co-activation network (MACM) and PET/SPECT density maps

	Region-of-Interest				
Mental Functions	Centromedial Amygdala	Precuneus	Intraparietal Sulcus	Angular Gyrus	Middle Temporal Gyrus
$5HT_{1A}$	0.207	0.195	0.173	0.199	0.359
$5HT_{1B}$	0.233	0.367	0.339	0.350	0.240
$5HT_{2A}$	0.141	0.299	0.263	0.278	0.300
$5HT_4$	0.282	0.123	0.116	0.132	0.158
$5HT_6$	0.229	0.342	0.303	0.320	0.304
5HTT	0.532	0.131	0.106	0.124	0.143
α4β2	0.231	0.208	0.219	0.232	0.114
CB_1	0.226	0.329	0.299	0.344	0.286
D1	0.396	0.241	0.193	0.230	0.235
D_2	0.436	0.152	0.141	0.166	0.129
DAT	0.420	0.125	0.119	0.136	0.098
GABA	0.141	0.271	0.240	0.246	0.252
H_3	0.474	0.243	0.222	0.252	0.171
M_1	0.184	0.302	0.253	0.274	0.254
$mGLUR_5$	0.196	0.345	0.295	0.329	0.314
MOR	0.385	0.294	0.278	0.352	0.275
NAT	0.253	0.217	0.215	0.234	0.109
NMDA	0.236	0.251	0.228	0.239	0.228
VAChT	0.446	0.116	0.112	0.132	0.090

Note. Spatial similarity was established using Pearson's correlation across 226,654 voxels. Statistical maps of mental processes derived from Hansen and colleagues (2022).

Supplementary Table 6. (Reactive Aggression) Spatial Similarity between Region-specific co-activation network (MACM) and Mental Processes

	Region-of-Interest			
Mental Functions	Centromedial Amygdala	Posterior Insula	Periaqueductal Grey	Central Opercular Cortex
Action	0.132	0.250	0.297	0.414
Auditory Perception	0.173	0.420	0.172	0.440
Cognitive Control	0.157	0.059	0.432	0.180
Face Detection	0.521	0.067	0.314	0.167
Language	0.214	0.101	0.255	0.196
Motivation	0.583	0.212	0.535	0.319
Multi Demand	0.199	0.140	0.423	0.283
Physiological Arousal	0.346	0.592	0.439	0.757
Social Inference	0.193	-0.026	-0.030	-0.061
Social Representation	0.308	0.003	0.119	0.022
Spatial Attention	-0.056	-0.058	0.026	-0.071
Spatial Memory	0.074	0.022	0.128	0.039
Valuation	0.178	0.045	0.091	0.037

Note. Spatial similarity was established using Pearson's correlation across 226,654 voxels. Statistical maps of mental processes derived from Dugré et Potvin (BioRxiv) and are available (https://neurovault.org/collections/13769/).

Supplementary Table 7. (Reactive Aggression) Spatial Similarity between Region-specific co-activation network (MACM) and PET/SPECT density maps

	Region-of-Interest			
Mental Functions	Centromedial Amygdala	Posterior Insula	Periaqueductal Grey	Central Opercular Cortex
$5HT_{1A}$	0.289	0.174	0.090	0.234
$5HT_{1B}$	0.213	0.218	0.219	0.294
$5HT_{2A}$	0.163	0.154	0.106	0.227
$5HT_4$	0.229	0.187	0.189	0.223
$5HT_6$	0.209	0.269	0.162	0.325
5HTT	0.474	0.363	0.572	0.371
α4β2	0.175	0.258	0.357	0.255
CB_1	0.218	0.215	0.142	0.314
D1	0.347	0.346	0.268	0.361
D_2	0.346	0.314	0.325	0.342
DAT	0.333	0.315	0.320	0.313
GABA	0.159	0.189	0.121	0.211
H_3	0.397	0.329	0.327	0.411
M_1	0.198	0.188	0.067	0.259
$mGLUR_5$	0.215	0.274	0.127	0.331
MOR	0.362	0.297	0.336	0.392
NAT	0.201	0.384	0.401	0.399
NMDA	0.222	0.257	0.244	0.260
VAChT	0.349	0.373	0.347	0.366

Note. Spatial similarity was established using Pearson's correlation across 226,654 voxels. Statistical maps of mental processes derived from Hansen and colleagues (2022).

Supplementary Table 8. (Proactive Aggression) Spatial Similarity between Region-specific co-activation network (MACM) and Mental Processes

	Region-of-Interest	
Mental Functions	Centromedial Amygdala	Basal Forebrain
Action	0.322	0.147
Auditory Perception	0.247	0.055
Cognitive Control	0.221	0.266
Face Detection	0.460	0.268
Language	0.206	0.126
Motivation	0.610	0.772
Multi Demand	0.272	0.326
Physiological Arousal	0.486	0.371
Social Inference	0.056	0.054
Social Representation	0.183	0.137
Spatial Attention	-0.064	0.046
Spatial Memory	0.095	0.105
Valuation	0.124	0.234

Note. Spatial similarity was established using Pearson's correlation across 226,654 voxels. Statistical maps of mental processes derived from Dugré et Potvin (BioRxiv) and are available (https://neurovault.org/collections/13769/).

Supplementary Table 9. (Proactive Aggression) Spatial Similarity between Region-specific co-activation network (MACM) and Mental Processes

	Region-of-Interest	
Mental Functions	Centromedial Amygdala	Basal Forebrain
$5HT_{1A}$	0.189	0.120
$5HT_{1B}$	0.251	0.247
$5HT_{2A}$	0.149	0.145
$5HT_4$	0.298	0.242
$5HT_6$	0.258	0.212
5HTT	0.539	0.544
α4β2	0.245	0.253
CB_1	0.246	0.172
D1	0.420	0.400
D_2	0.469	0.455
DAT	0.449	0.434
GABA	0.150	0.124
H_3	0.505	0.471
M_1	0.197	0.176
$mGLUR_5$	0.209	0.179
MOR	0.398	0.466
NAT	0.278	0.264
NMDA	0.247	0.213
VAChT	0.479	0.405

Note. Spatial similarity was established using Pearson's correlation across 226,654 voxels. Statistical maps of mental processes derived from Hansen and colleagues (2022).

Supplementary Table 10. (Physical Aggression) Spatial Similarity between Region-specific co-activation network (MACM) and Mental Processes

	Region-of-Interest		
Mental Functions	dorsal Premotor Cortex	dorsal Caudate	dorsal Anterior Cingulate Cortex
Action	0.555	0.400	0.356
Auditory Perception	0.148	0.189	0.155
Cognitive Control	0.749	0.552	0.640
Face Detection	0.218	0.236	0.238
Language	0.535	0.392	0.456
Motivation	0.218	0.445	0.436
Multi Demand	0.546	0.509	0.688
Physiological Arousal	0.270	0.446	0.499
Social Inference	-0.024	-0.016	-0.018
Social Representation	0.185	0.148	0.160
Spatial Attention	0.148	0.047	0.043
Spatial Memory	0.212	0.123	0.128
Valuation	0.046	0.069	0.147

Note. Spatial similarity was established using Pearson's correlation across 226,654 voxels. Statistical maps of mental processes derived from Dugré et Potvin (BioRxiv) and are available (https://neurovault.org/collections/13769/).

Supplementary Table 11. (Physical Aggression) Spatial Similarity between Region-specific co-activation network (MACM) and Mental Processes

	Region-of-Interest		
Mental Functions	dorsal Premotor Cortex	dorsal Caudate	dorsal Anterior Cingulate Cortex
$5HT_{1A}$	0.159	0.076	0.169
$5HT_{1B}$	0.333	0.246	0.318
$5HT_{2A}$	0.254	0.140	0.235
$5HT_{4}$	0.113	0.272	0.145
$5HT_{6}$	0.289	0.331	0.298
5HTT	0.105	0.301	0.230
α4β2	0.211	0.290	0.250
CB_{1}	0.288	0.206	0.281
D1	0.179	0.384	0.273
D_{2}	0.136	0.457	0.222
DAT	0.119	0.405	0.216
GABA	0.218	0.136	0.194
H_{3}	0.250	0.367	0.321
M_{1}	0.237	0.193	0.217
$mGLUR_{5}$	0.281	0.219	0.293
MOR	0.272	0.360	0.398
NAT	0.211	0.222	0.237
NMDA	0.210	0.232	0.227
VAChT	0.125	0.411	0.202

Note. Spatial similarity was established using Pearson's correlation across 226,654 voxels. Statistical maps of mental processes derived from Hansen and colleagues (2022).

Supplementary Table 12. (Verbal Aggression) Spatial Similarity between Region-specific co-activation network (MACM) and Mental Processes

Mental Functions	Region-of-Interest
	dorsal Anterior Cingulate Cortex
Action	0.355
Auditory Perception	0.157
Cognitive Control	0.641
Face Detection	0.237
Language	0.454
Motivation	0.440
Multi Demand	0.689
Physiological Arousal	0.503
Social Inference	-0.019
Social Representation	0.156
Spatial Attention	0.049
Spatial Memory	0.129
Valuation	0.149

Note. Spatial similarity was established using Pearson's correlation across 226,654 voxels. Statistical maps of mental processes derived from Dugré et Potvin (BioRxiv) and are available (https://neurovault.org/collections/13769/).

Supplementary Table 13. (Verbal Aggression) Spatial Similarity between Region-specific co-activation network (MACM) and Mental Processes

Mental Functions	Region-of-Interest
	dorsal Anterior Cingulate Cortex
$5HT_{1A}$	0.172
$5HT_{1B}$	0.321
$5HT_{2A}$	0.237
$5HT_4$	0.146
$5HT_6$	0.299
5HTT	0.231
α4β2	0.253
CB_1	0.285
D1	0.276
D_2	0.222
DAT	0.215
GABA	0.196
H_3	0.325
M_1	0.219
$mGLUR_5$	0.295
MOR	0.402
NAT	0.239
NMDA	0.229
VAChT	0.203

Note. Spatial similarity was established using Pearson's correlation across 226,654 voxels. Statistical maps of mental processes derived from Hansen and colleagues (2022).

6. Supplementary Figures

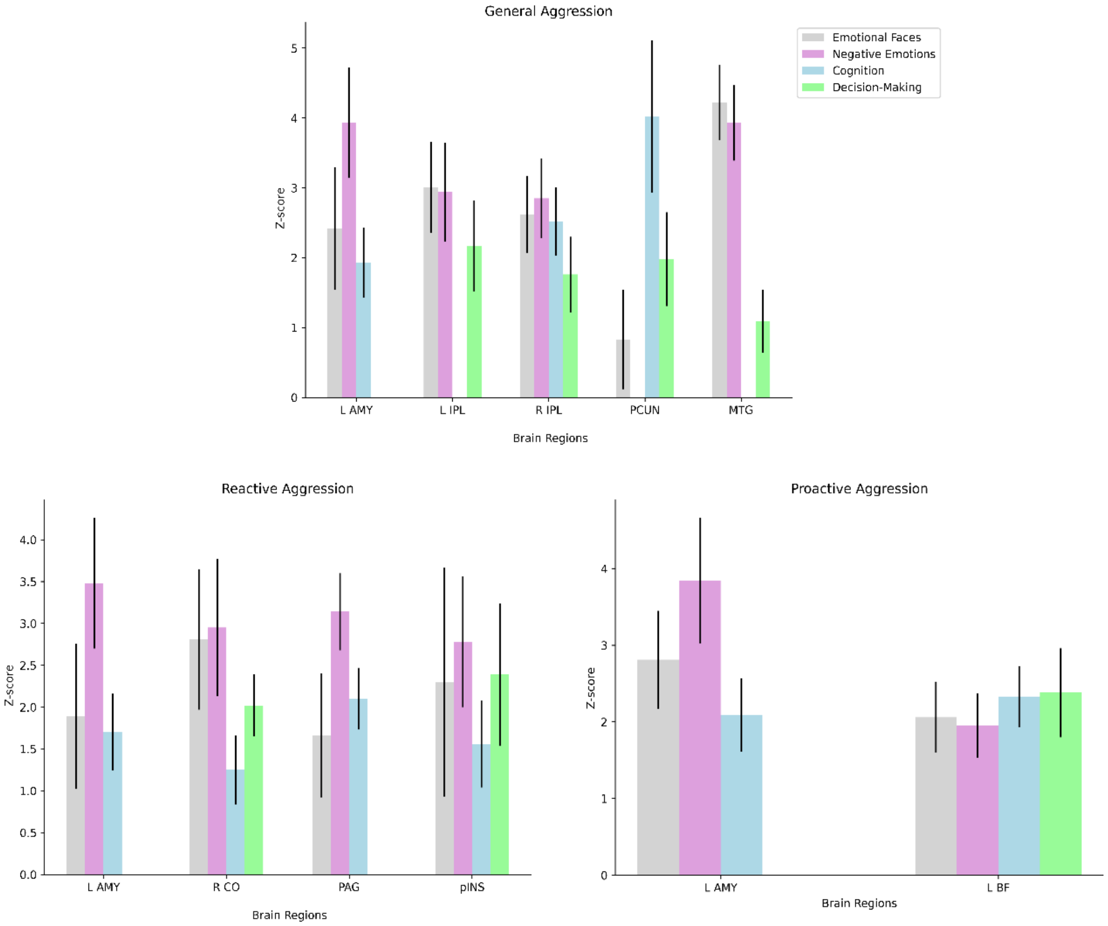

Supplementary Figure 1. Subanalyses showing the effects of fMRI Task domains on brain activity associated with Aggression. These bar graphs represent the average Z-score across voxels of each brain regions identified in the main meta-analysis. Experiments were manually annotated if they reported a task involving Emotional Faces (e.g., fear, sad, happy), Negative Emotions (e.g., anger-scripts, passive viewing, anger induction), Cognition (e.g., stroop task, go/no-go, n-back), and Decision-Making (e.g., Monetary Incentive Delay Task, Colorado Balloon Game). General Aggression included 23 experiments for Emotional Faces, 30 experiments for Negative Stimuli, 17 experiments for Cognition, and 15 experiments on Decision-Making. Reactive Aggression included 25 experiments on Emotional Faces, 36 experiments for Negative Emotions, 14 experiments for Cognition and 11 experiments for Decision-Making. Proactive Aggression included 8 experiments for Emotional Faces, 5 experiments for Negative Emotions, 6 experiments for Cognition, and 11 experiments for Decision-Making. L = Left, R = Right, AMY = Amygdala, IPL = Inferior Parietal Lobule, PCUN = Precuneus, MTG = Middle Temporal Gyrus, CO = Central Opercular, PAG = Periaqueductal Grey, pINS = Posterior Insula, BF = Basal Forebrain/Septal Area.

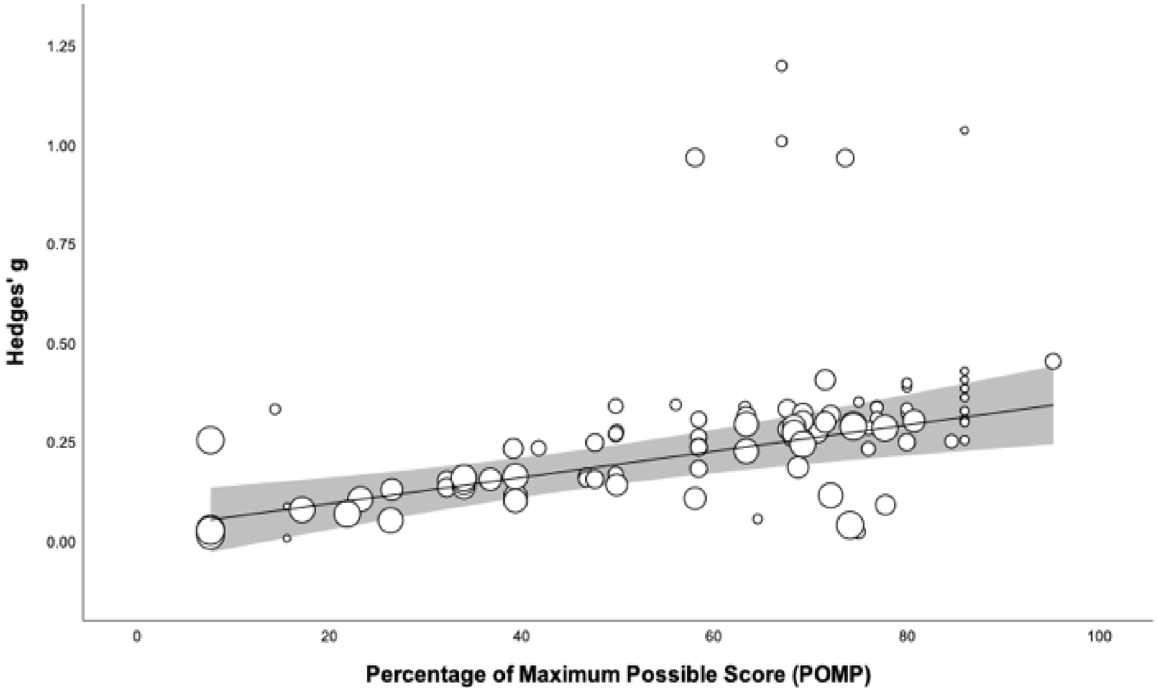

Supplementary Figure 2. Bubble plot representing the meta-regression results on the association between **General Aggression (POMP score)** and the **Left Inferior Temporal Gyrus**. The size of the bubble is proportional to the inverse variance.

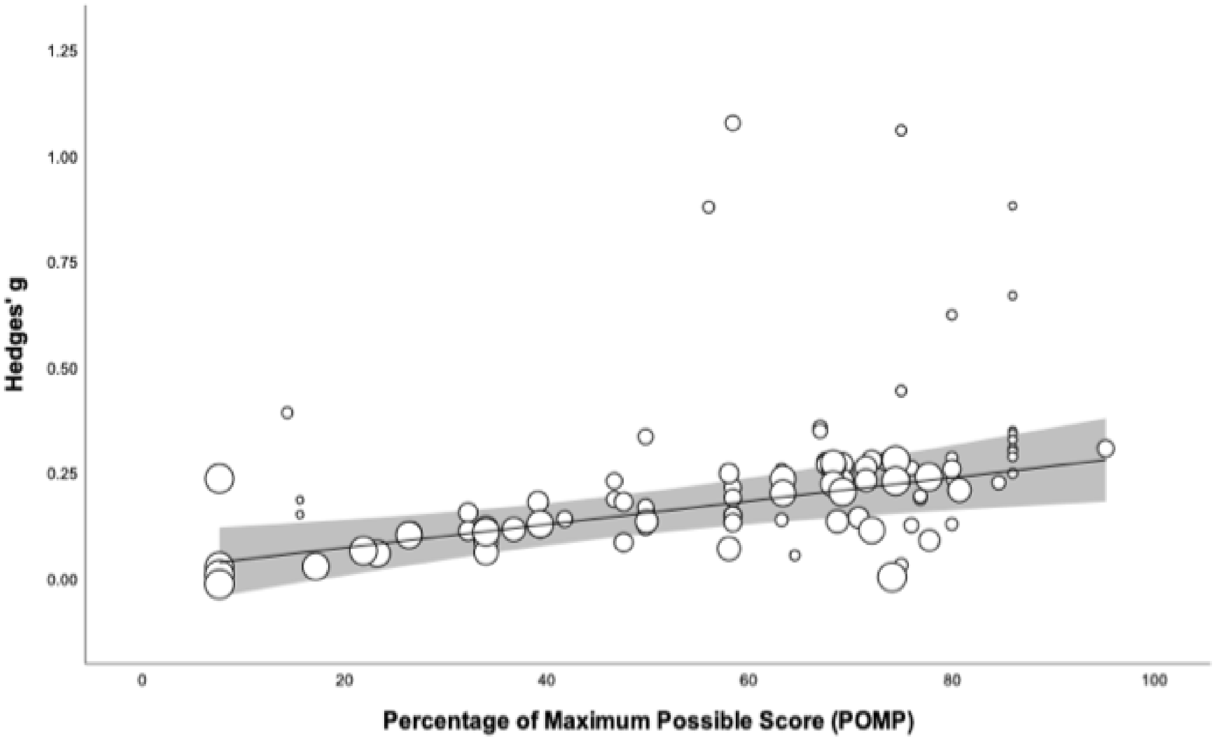

Supplementary Figure 3. Bubble plot representing the meta-regression results on the association between **General Aggression (POMP score)** and the **Precuneus**. The size of the bubble is proportional to the inverse variance.

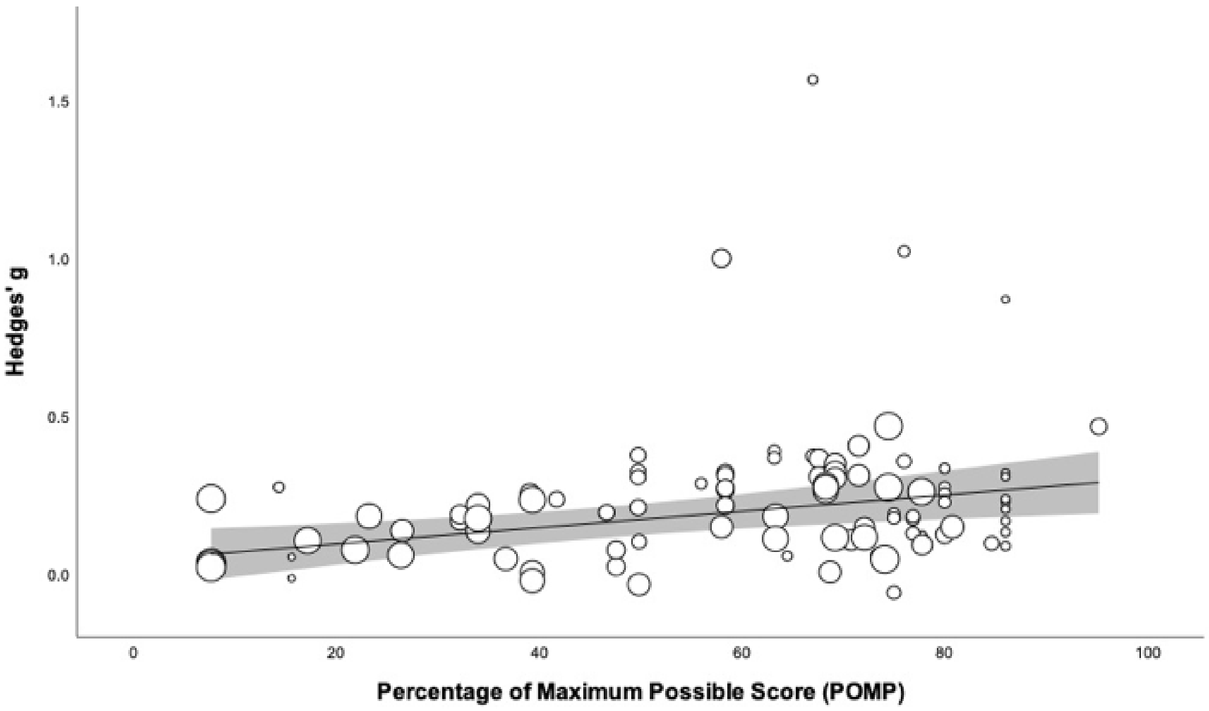

Supplementary Figure 4. Bubble plot representing the meta-regression results on the association between **General Aggression (POMP score)** and the **Right Inferior Temporal Gyrus.** The size of the bubble is proportional to the inverse variance.

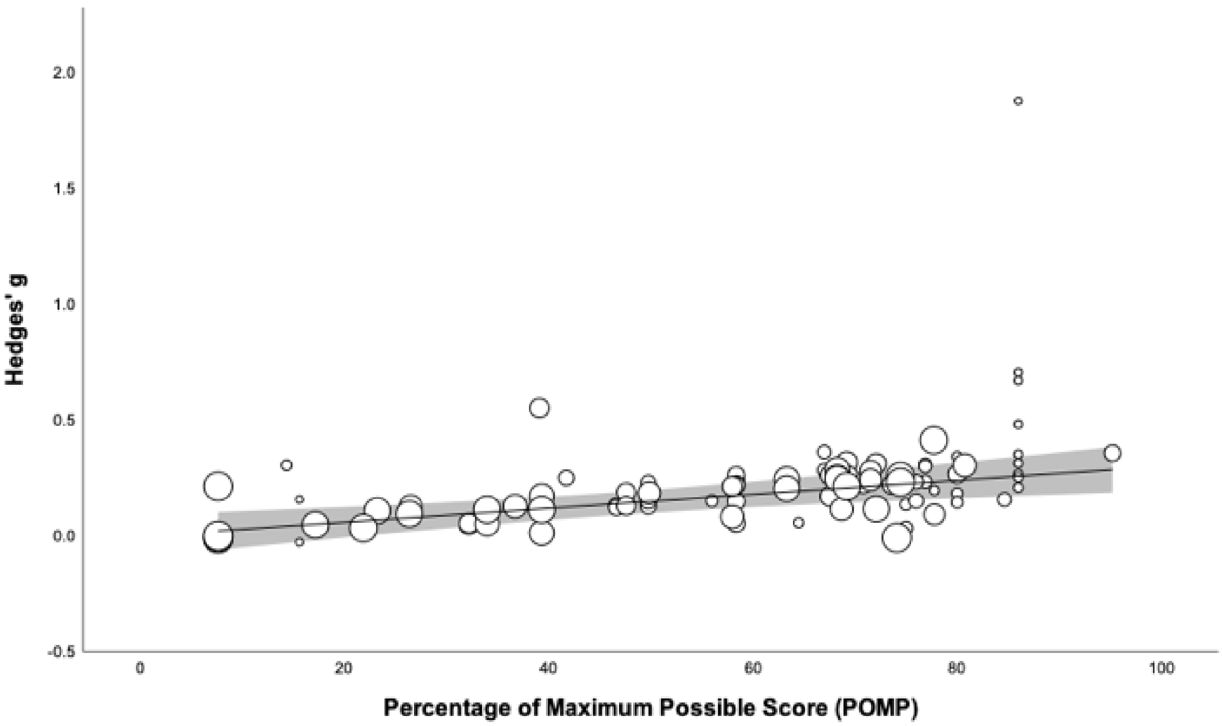

Supplementary Figure 5. Bubble plot representing the meta-regression results on the association between **General Aggression (POMP score)** and the **Secondary Visual Cortex.** The size of the bubble is proportional to the inverse variance.

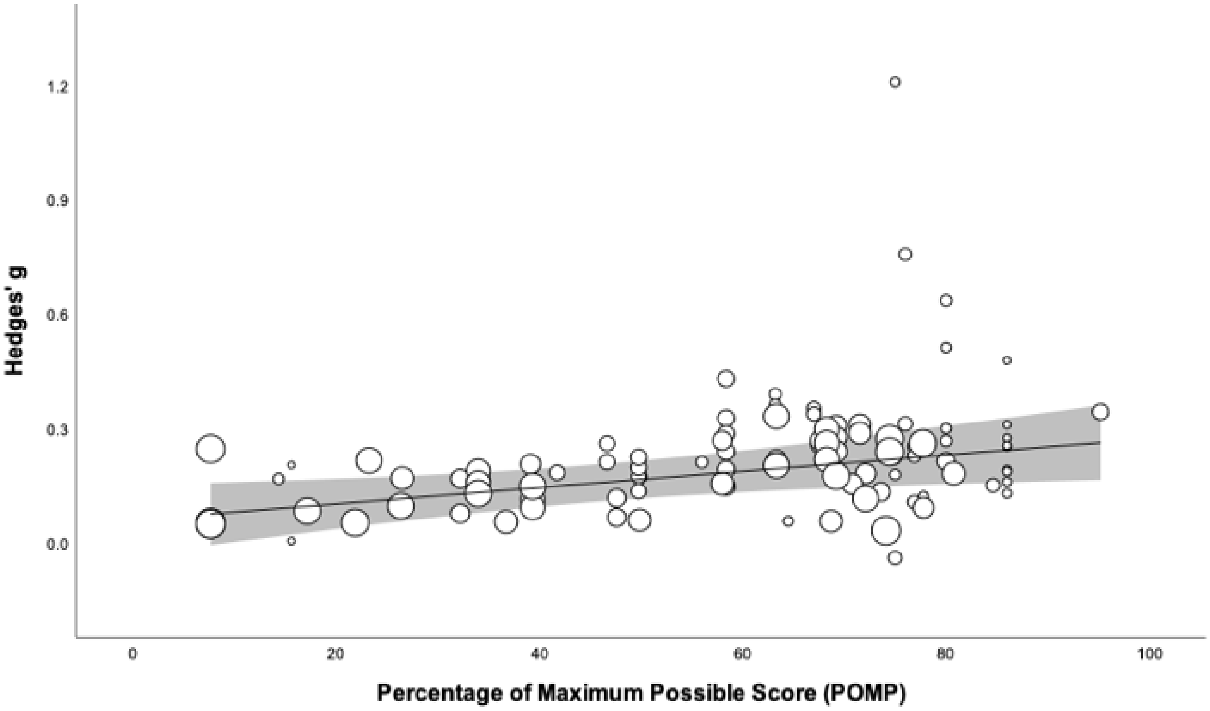

Supplementary Figure 6. Bubble plot representing the meta-regression results on the association between **General Aggression (POMP score)** and the **Right Angular Gyrus.** The size of the bubble is proportional to the inverse variance.

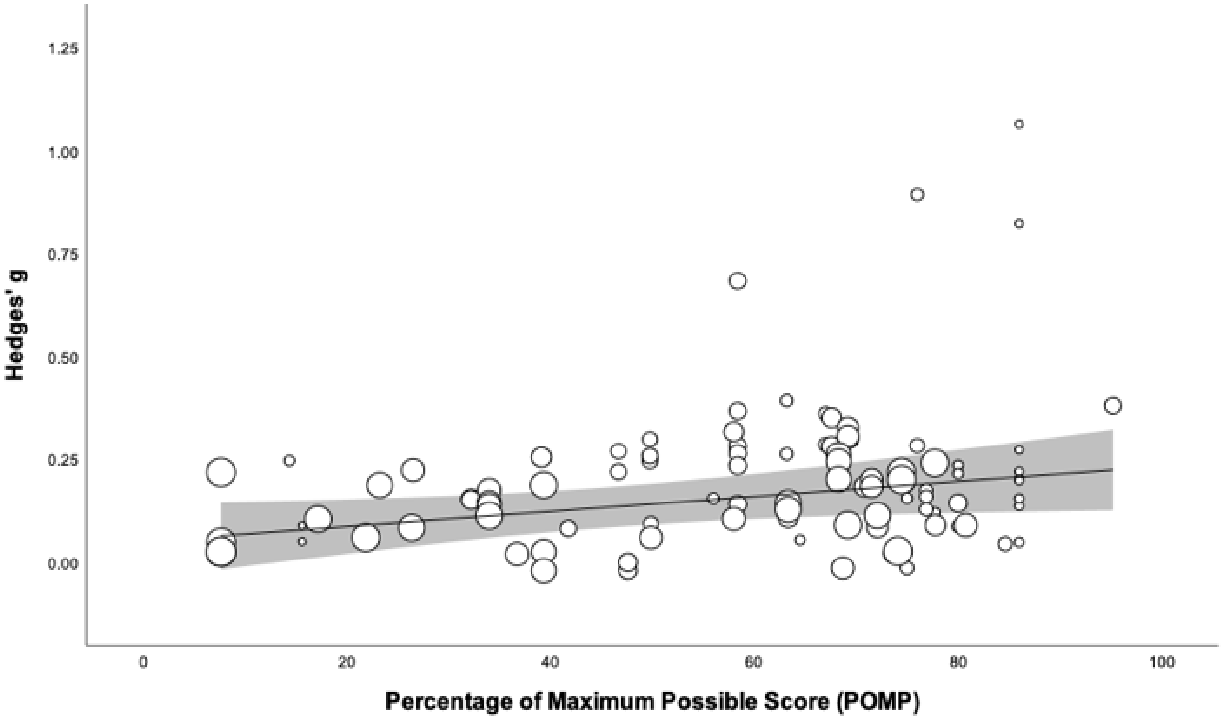

Supplementary Figure 7. Bubble plot representing the meta-regression results on the association between **General Aggression (POMP score)** and the **Left Crus II.** The size of the bubble is proportional to the inverse variance.

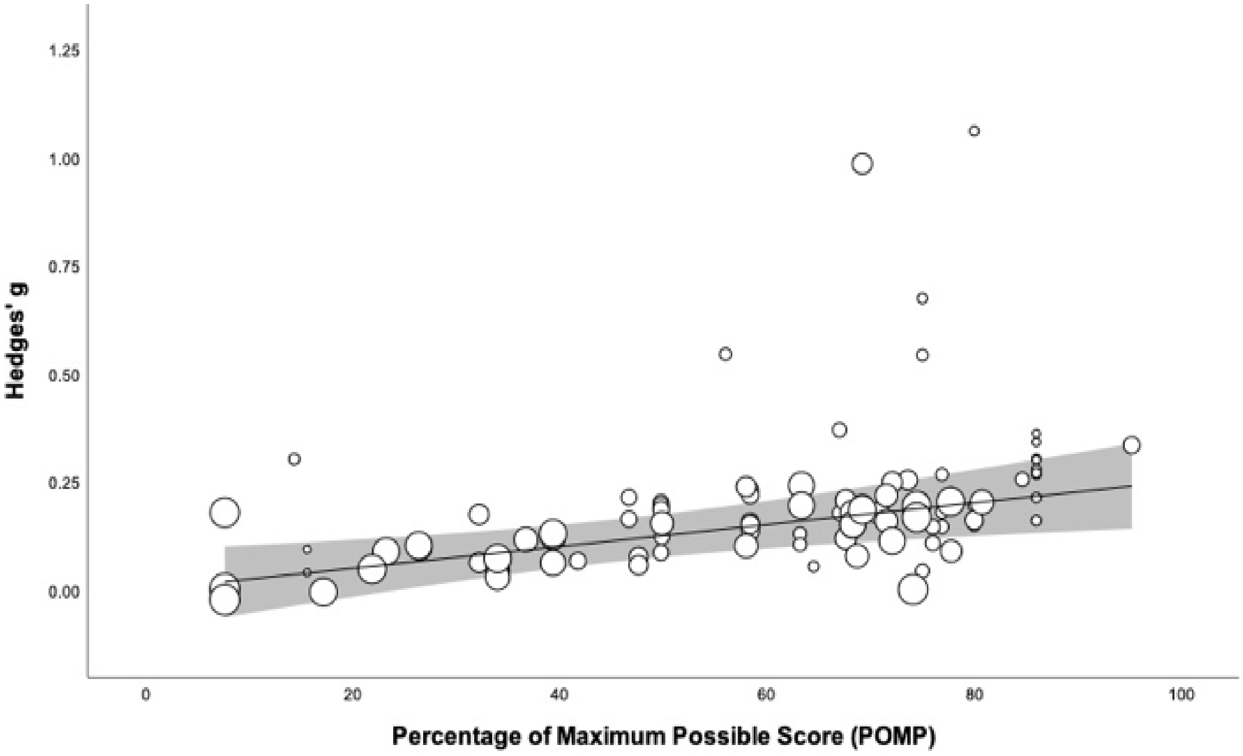

Supplementary Figure 8. Bubble plot representing the meta-regression results on the association between **General Aggression (POMP score)** and the **Left Premotor Cortex.** The size of the bubble is proportional to the inverse variance.

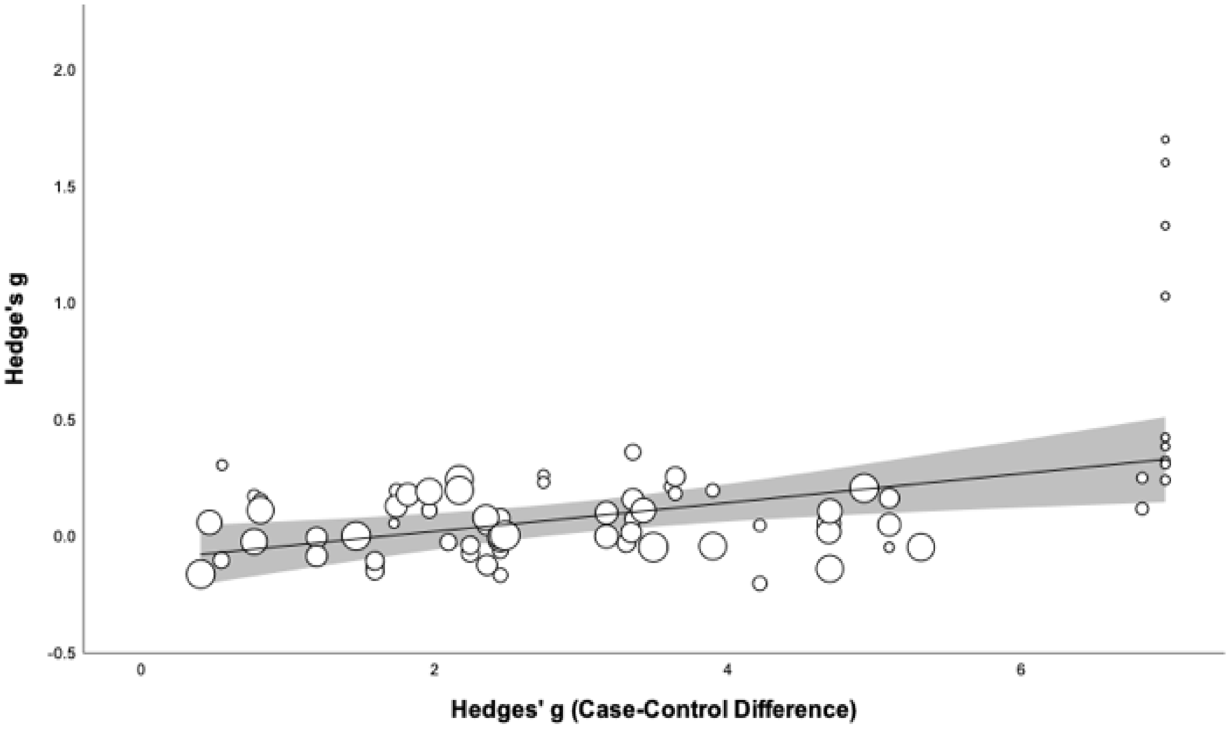

Supplementary Figure 9. Bubble plot representing the meta-regression results on the association between **General Aggression (Hedges' g)** and the **Left Lateral Occipital Gyrus.** The size of the bubble is proportional to the inverse variance.

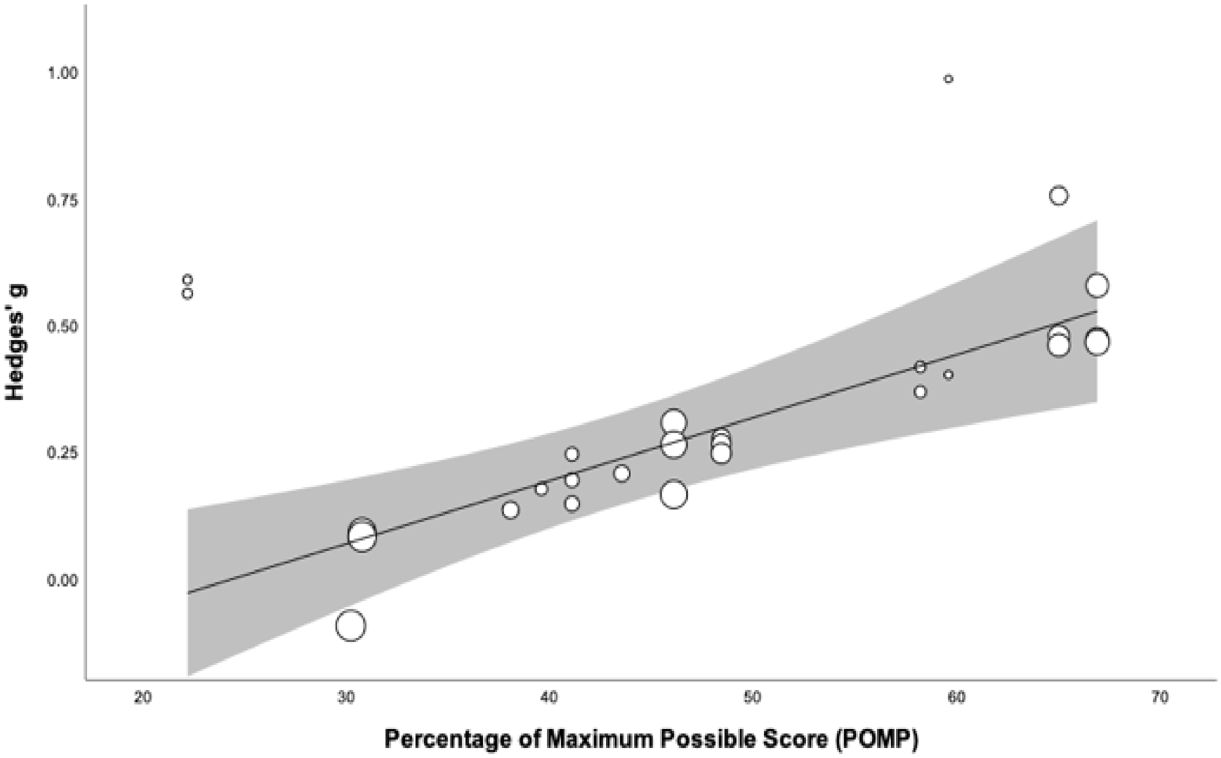

Supplementary Figure 10. Bubble plot representing the meta-regression results on the association between **Physical Aggression (POMP Score)** and the **Secondary Visual Area.** The size of the bubble is proportional to the inverse variance.

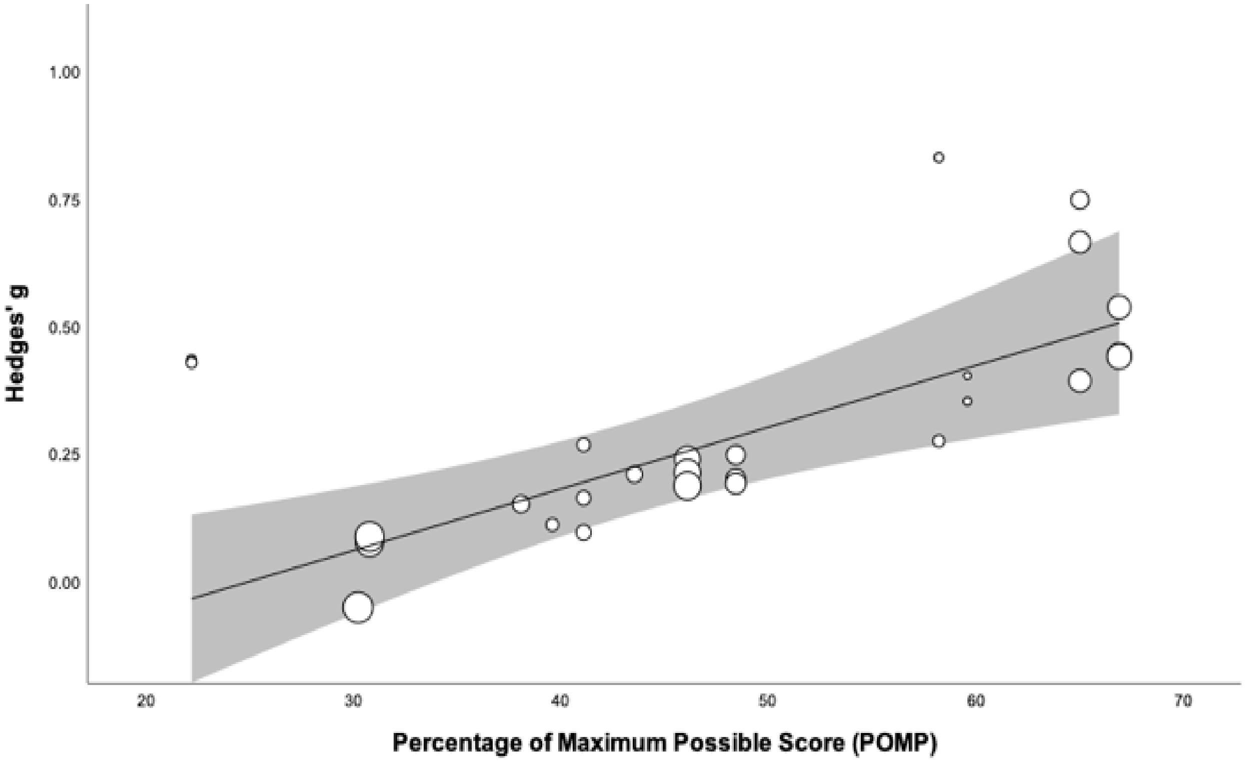

Supplementary Figure 11. Bubble plot representing the meta-regression results on the association between **Physical Aggression (POMP Score)** and the **Dorsal Anterior Cingulate Cortex.** The size of the bubble is proportional to the inverse variance.

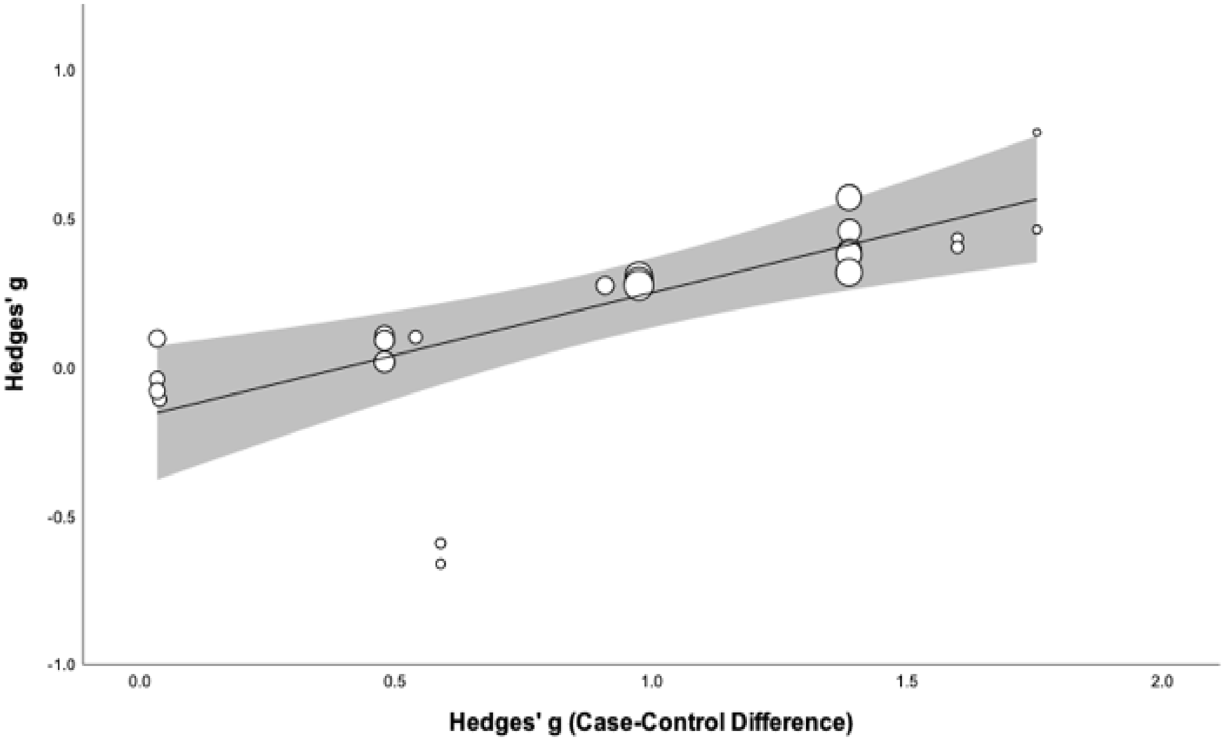

Supplementary Figure 12. Bubble plot representing the meta-regression results on the association between **Verbal Aggression (Hedges' g)** and the **Visual Cortex (V3).** The size of the bubble is proportional to the inverse variance.

REFERENCES

1. Eickhoff SB, Bzdok D, Laird AR, Kurth F, Fox PT. Activation likelihood estimation meta-analysis revisited. *Neuroimage* 2012; **59**(3)**:** 2349-2361.

2. Eickhoff SB, Laird AR, Grefkes C, Wang LE, Zilles K, Fox PT. Coordinate-based activation likelihood estimation meta-analysis of neuroimaging data: a random-effects approach based on empirical estimates of spatial uncertainty. *Hum Brain Mapp* 2009; **30**(9)**:** 2907-2926.

3. Albajes-Eizagirre A, Solanes A, Vieta E, Radua J. Voxel-based meta-analysis via permutation of subject images (PSI): Theory and implementation for SDM. *Neuroimage* 2019; **186:** 174-184.

4. Dugré JR, Radua J, Carignan-Allard M, Dumais A, Rubia K, Potvin S. Neurofunctional abnormalities in antisocial spectrum: A meta-analysis of fMRI studies on Five distinct neurocognitive research domains. *Neurosci Biobehav Rev* 2020; **119:** 168-183.

5. Radua J, Mataix-Cols D, Phillips ML, El-Hage W, Kronhaus DM, Cardoner N *et al.* A new meta-analytic method for neuroimaging studies that combines reported peak coordinates and statistical parametric maps. *Eur Psychiatry* 2012; **27**(8)**:** 605-611.

6. Albajes-Eizagirre A, Solanes A, Radua J. Meta-analysis of non-statistically significant unreported effects. *Stat Methods Med Res* 2019; **28**(12)**:** 3741-3754.

7. Salo T, Yarkoni, T., Nichols, T.E., Poline, J-B., Bigel, M., Bottenhorn, K.L. NiMARE: Neuroimaging Meta-Analysis Research Environment. *NeuroLibre Reproducible Preprint Server* 2022; **1**(1).

8. Dugré JR, Potvin S. Towards a Neurobiologically-driven Ontology of Mental Functions: A Data-driven Summary of the Twenty Years of Neuroimaging Meta-Analyses. 2023**:** 2023.2003.2029.534795.

9. Hansen JY, Shafiei G, Markello RD, Smart K, Cox SML, Nørgaard M *et al.* Mapping neurotransmitter systems to the structural and functional organization of the human neocortex. *Nat Neurosci* 2022; **25**(11)**:** 1569-1581.

10. Webster GD, DeWall CN, Pond Jr RS, Deckman T, Jonason PK, Le BM *et al.* The brief aggression questionnaire: Structure, validity, reliability, and generalizability. 2015; **97**(6)**:** 638-649.

11. Buss AH, Durkee A. An inventory for assessing different kinds of hostility. *J Consult Psychol* 1957; **21**(4)**:** 343-349.

12. Buss AH, Perry M. The aggression questionnaire. *J Pers Soc Psychol* 1992; **63**(3)**:** 452-459.

13. Buss AH, Warren W. *Aggression questionnaire:(AQ)*. Western Psychological Services Torrence, CA2000.

14. Achenbach TM, Rescorla LA. *Manual for the ASEBA school-age forms & profiles: child behavior checklist for ages 6-18, teacher's report form, youth self-report: an integrated system of multi-informant assessment*. University of Vermont, research center for children youth & families2001.

15. Dodge KA, Coie JD. Social-information-processing factors in reactive and proactive aggression in children's peer groups. *J Pers Soc Psychol* 1987; **53**(6)**:** 1146-1158.

16. Raine A, Dodge K, Loeber R, Gatzke-Kopp L, Lynam D, Reynolds C *et al.* The Reactive-Proactive Aggression Questionnaire: Differential Correlates of Reactive and Proactive Aggression in Adolescent Boys. *Aggress Behav* 2006; **32**(2)**:** 159-171.

17. Spielberger CD, Sydeman SJ, Owen AE, Marsh BJ. *Measuring anxiety and anger with the State-Trait Anxiety Inventory (STAI) and the State-Trait Anger Expression Inventory (STAXI)*. Lawrence Erlbaum Associates Publishers1999.

18. Coccaro EF, Berman ME, Kavoussi RJJPr. Assessment of life history of aggression: development and psychometric characteristics. 1997; **73**(3)**:** 147-157.

19. Drislane LE, Patrick CJ, Arsal G. Clarifying the content coverage of differing psychopathy inventories through reference to the triarchic psychopathy measure. *Psychol Assess* 2014; **26**(2)**:** 350-362.

20. Hampel R, Selg H. *Fragebogen zur Erfassung von Aggressivitätsfaktoren: FAF*. Hogrefe1975.

21. Heubrock D, Petermann F. *Kurzfragebogen zur Erfassung von Aggressivitätsfaktoren: K-FAF*. Hogrefe2008.

22. Crowley TJ, Mikulich SK, Ehlers KM, Whitmore EA, MacDonald MJ. Validity of structured clinical evaluations in adolescents with conduct and substance problems. *J Am Acad Child Adolesc Psychiatry* 2001; **40**(3)**:** 265-273.

23. Overall JE, Gorham DRJPb. The Brief Psychiatric Rating Scale (BPRS): recent developments in ascertainment and scaling. 1988.

24. Gunn J, Robertson GJBJC. Drawing a criminal profile. 1976; **16:** 156.

25. Espelage DL, Holt MK. Bullying and victimization during early adolescence: Peer influences and psychosocial correlates. *Journal of Emotional Abuse* 2001; **2**(2-3)**:** 123-142.

26. Lilienfeld SO, Andrews BPJJopa. Development and preliminary validation of a self-report measure of psychopathic personality traits in noncriminal population. 1996; **66**(3)**:** 488-524.
